# The Puzzling *Falcomurus* Mandal (Collembola, Orchesellidae, Heteromurinae): A Review

**DOI:** 10.3390/insects12070650

**Published:** 2021-07-16

**Authors:** Bruno C. Bellini, Paolla G. C. de Souza, Penelope Greenslade

**Affiliations:** 1 Laboratório de Collembola, Departamento de Botânica e Zoologia, Centro de Biociências, Campus Universitário, Universidade Federal do Rio Grande do Norte—UFRN. BR 101, Lagoa Nova, Natal 59072-970, Brazil; paollasouzac@gmail.com; 2 School of Science, Psychology and Sports, Federation University, Ballarat, VIC 3353, Australia; 3 Department of Biology, Australian National University, GPO Box, Canberra, ACT 0200, Australia

**Keywords:** chaetotaxy, Entomobryoidea, Entomobryomorpha, Heteromurini, taxonomy

## Abstract

**Simple Summary:**

Springtails are tiny microarthropods found mainly in soil habitats around the globe. *Falcomurus* is a genus of Heteromurinae (Orchesellidae), currently with a single species from India. Here, we revise the genus, transferring *Dicranocentrus litoreus* Mari-Mutt and *D. halophilus* Mari-Mutt to *Falcomurus* and describing two new taxa from marine littoral habitats in Australian archipelagos. We discovered the morphology of *Falcomurus* is quite similar among its species, but some characters of its chaetotaxy (the distribution and morphology of body chaetae) are useful to clearly separate them from each other.

**Abstract:**

*Falcomurus* Mandal is currently a monotypic genus of Heteromurinae described from India in 2018. Its key characters are the first antennal segment subdivided, the second undivided and the third annulated; the first abdominal segment lacking macrochaetae; and the presence of a sinuous modified macrochaeta on the proximal dens. Some details of its morphology were recently put in doubt, and so its genus status and affinities remain uncertain. Here, we revise the genus based on the type material of *Dicranocentrus litoreus* Mari-Mutt, as well as provide the description of two new species from Australian archipelagos and a reinterpretation of the chaetotaxy of *Falcomurus chilikaensis* Mandal and *D. halophilus* Mari-Mutt. After our revision, *Falcomurus* shows a well-conserved chaetotaxy and overall morphology, which allowed us to provide an updated generic diagnosis. While the antennae morphology of *Falcomurus* resembles that of *Dicranocentrus* Schött, its dorsal sensillar and macrochaetotaxy suggest it is closely related to *Heteromurus* Wankel, as originally stated by Mandal. The main features useful to separate *Falcomurus* species are the head, mesothorax and fourth abdominal segment chaetotaxy. We also provide a key to its five species, a comparative table and notes on the affinities and distribution of *Falcomurus*.

## 1. Introduction

Recent studies reviewing the systematics within the Entomobryoidea have improved the understanding of the relationships between the families, subfamilies, tribes and genera, plus identified new more reliable diagnostic characters [1,2,3]. In particular, the affinities and internal organisation of the Orchesellinae sensu Soto-Adames et al. [4] were revised, and this taxon was reinstated as a full family, as earlier proposed [3,5]. Other studies have provided more evidence to support the validity and possible relationships within the Orchesellidae and suggested the sensillar pattern of the trunk segments may better distinguish its lineages [3,6,7,8].

Among the Orchesellidae, the Heteromurinae are defined by the presence of coarsely ciliate scales on the body and a sensillar formula from the dorsal mesothorax to the third abdominal segment of 2,2|1,3,3 [3]. The most recent revisions dealing with the Heteromurinae subdivided it into two tribes and seven genera: *Mastigoceras* Handschin, 1924 [9] (the sole genus in Mastigocerini); and *Alloscopus* Börner, 1906 [10], *Dicranocentrus* Schött, 1893 [11], *Heteromurtrella* Mari-Mutt, 1979 [12], *Heteromurus* Wankel, 1860 [13], *Pseudodicranocentrus* Mari-Mutt, 1981 [14] and *Sinodicranocentrus* Zhang, 2020 [8] (all from Heteromurini) [1,3,8]. In [8], the status of the recently described *Falcomurus* Mandal, 2018 [15] in the Heteromurini was disregarded, because of mistakes in its description and similarities with other taxa.

*Falcomurus* is currently a monotypic genus of Heteromurini from India, with many similarities to *Heteromurus*. Its most striking character is the presence of a sinuous ciliate macrochaeta on the proximal dens [15]. The original description of its dorsal chaetotaxy does not have a clear match among the Heteromurini, with the exception of an absence of macrochaetae on the first abdominal segment, recorded only for *Heteromurus*, including its subgenus *Verhoeffiela* Absolon, 1900 [16], and two species of *Dicranocentrus*, *D. litoreus* Mari-Mutt, 1985 and *D. halophilus* Mari-Mutt, 1985 [8,15,17]. These *Dicranocentrus* species actually show more similarities with *Heteromurus* concerning their dorsal chaetotaxy and also present the same sinuous dental macrochaeta described in *Falcomurus* [17] (p. 313, Figure 69 and pp. 318–320).

Unlike the Northern Hemisphere collembolan faunas, the Orchesellidae fauna of Australia is represented by few species in few genera, all with limited distributions. There is only one species of *Heteromurus*, *H. major* (Moniez, 1889) [18], introduced and only found in very disturbed habitats, such as home gardens or public parks. The common, abundant and widespread genus *Orchesella* Templeton, 1836 [19] of the Northern Hemisphere, is not present. The genera that are present are *Alloscopus*, *Australotomurus* Stach, 1947 [20] and *Isotobrya* Womersley, 1934 [21], with one, seven and two species, respectively [22]. The species in each of the genera live in different habitats, such as soil, coastal and montane heathland and termite nests, respectively. Both *Australotomurus* and *Isotobrya* are endemic to Australia and it appears they are representatives of a more ancient, now relict, fauna than the present dominant genus of Entomobryoidea, *Entomobrya* Rondani, 1861 [23].

Here, we provide a review of *Falcomurus* based on the literature, a revision of the *Dicranocentrus litoreus* type material and the description of two new Australian species from tropical marine littoral habitats. Using a comparative morphological analysis, we reinterpreted the dorsal chaetotaxy and other morphological features of its type species. A revised generic diagnosis, discussion on the generic affinities, key to its species and a comparative table of their main features are also presented.

## 2. Materials and Methods

Specimens of the new species were mounted on glass slides in Berlese’s medium. Morphological studies and raw drawings were made with a Leica DM750 microscope with an attached drawing tube. Photographs were taken with a Leica MC170 HD camera attached to the microscope, using LAS 4.12 software. The final figures were digitized and organized in plates using CorelDraw X8 software. The type series is deposited at the Museum of Victoria, Melbourne, Australia, under the acronym of MVMA.

The chaetotaxy terminology used in this study mainly follows Fjellberg [24] for labial palp papillae; Gisin [25] for labial chaetotaxy, with additions of Zhang and Pan [26] and using Bellini et al. [27] as a model; Cipola et al. [28] for labral chaetotaxy; Mari-Mutt [29] for dorsal head chaetotaxy, with additions of Soto-Adames [30] and Zhang et al. [8]; Szeptycki [31] and Zhang and Deharveng [1] for S-chaetotaxy; and Szeptycki [32] for dorsal chaetotaxy, with additions and modifications provided by Soto-Adames [30], Cipola et al. [33] and Zhang et al. [3,8,34], using also Bellini et al. [27] as a model.

The abbreviations used in the descriptions are Abd.—abdominal segment(s); Ant.—antennal segment(s); mac—macrochaeta(e); mes—mesochaeta(e); mic—microchaeta(e); ms—S-microchaeta(e); sens—ordinary S-chaeta(e); and Th.—thoracic segment(s). Antennal segments I and II’s subdivisions are ‘a’ for the proximal subarticle and ‘b’ for the distal one.

The symbols used in the drawings to represent the dorsal chaetotaxy patterns are large blank circles for mac; large black circles for mes; small black circles for mic; blank circles with a small black circle inside for mac or mic; black chaetae-like drawings for sens or ms; black circles crossed by a line for pseudopores; ciliate chaetae-like symbols for bothriotricha; and a dash above or under any symbol for chaetae presence or absence. Taxonomic descriptions and comparisons were based on a half body. Chaetae labels (including rows/series) and labial papillae are marked in bold throughout the text.

## 3. Results

### 3.1. Taxonomic Summary and Genus Diagnosis

- Order Entomobryomorpha Börner, 1913 [35]
- Superfamily Entomobryoidea Womersley, 1934 [21]
- Family Orchesellidae Börner, 1906 [10], sensu Zhang et al. 2019 [3]
- Subfamily Heteromurinae Absolon and Ksenemann, 1942 [36], sensu Zhang and Deharveng, 2015 [1]
- Tribe Heteromurini Absolon and Ksenemann, 1942 [36], sensu Zhang et al. 2020 [8]
- Genus *Falcomurus* Mandal, 2018 [15]

**Figure 1:**
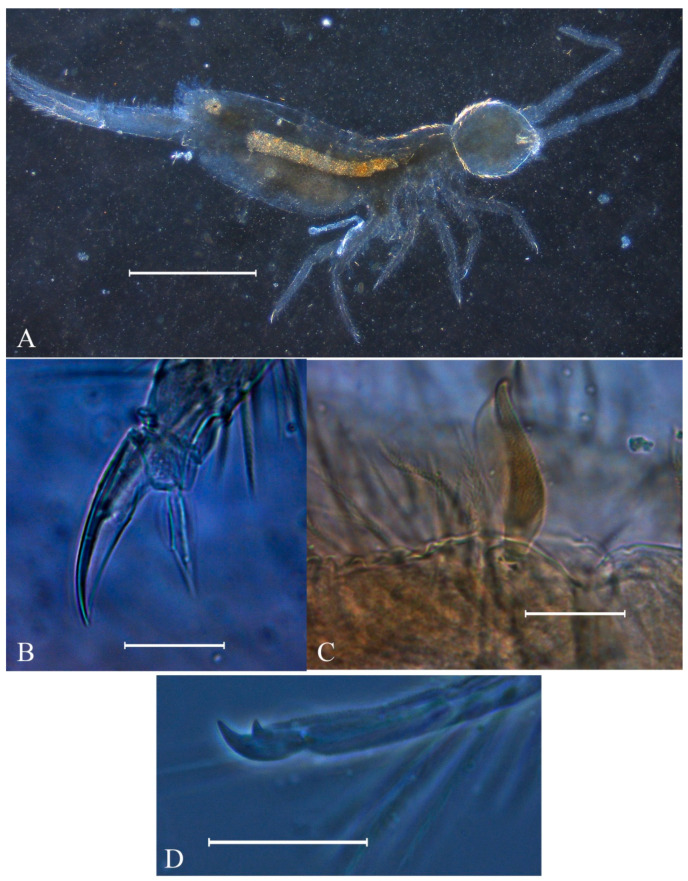
Photographs of the *Falcomurus* features: (**A**) habitus of *F. pulukokos* sp. nov. holotype (on slide), scale bar = 500 μm; (**B**) hind foot complex of *F. pulukokos* sp. nov., scale bar = 25 μm; (**C**) sinuous macrochaeta on dorso-proximal dens of *F. pulukokos* sp. nov., scale bar = 25 μm; (**D**) distal dens and mucro of *F. pulukokos* sp. nov., scale bar = 25 μm.

Diagnosis: Habitus similar to *Dicranocentrus* (Figure 1A). Apically rounded or truncate scales present at least over Ant. I, head, trunk and ventral furca. Ant. I subdivided in Ia and Ib, Ant. II subdivided or undivided, Ant. III and IV annulated. Dorsal head with at least **A0**, **A2**–**3**, **A6**, **M1**–**2**, **S1**, **S3**–**6** (including **S6i**), **Pa5**, **Pm3** and **Pp3** mac; **A1**, **M3**–**4**, **S0**, **S2** mac always absent. Prelabral chaetae normal (not bifurcate) and smooth, at least the labral **a1** chaeta enlarged. Eyes 8 + 8, postantennal organ absent. Tergal S-chaetotaxy from Th. II to Abd. V as 2,2|1,3,3,+,3 for sens, and 1,0|1,0,1,0,0 for ms. Trunk dorsal macrochaetotaxy reduced, Th. II with at least **a5**, **m4**–**4i**, **p1**–**3** (including **p2e**) mac; Th. III main mac as **p1**–**3** (including **p2e**) and **a5**; Abd. I devoid of mac; Abd. II mac as **m3**–**3e**, **m5**; Abd. III main mac as **m3** and **pm6**, **p6** mac possibly present in all species; Abd. IV with 2–3 central mac (**A3**, **B5** and **A6**, the latter also as mes). Ungues short and thick, internally with only the basal paired teeth, unguiculi excavated (Figure 1B). Proximal dens with a typically falcate (sinuous) ciliate mac (Figure 1C), dental spines absent, mucro bidentate, lacking the mucronal spine (Figure 1D) (adapted from [15,17]).

Type species: *Falcomurus chilikaensis* Mandal, 2018.

Remarks on the genus: *Falcomurus* was first described by [15] as a monotypic genus from India. However, two other species described earlier, *Dicranocentrus litoreus*, from the Philippines, and *D. halophilus* from Papua New Guinea, mostly comply with Mandal’s description, with the exception of the absence of **Ps2** mac on the dorsal head and presence of an unguiculus outer tooth, and are herein transferred to *Falcomurus*.

As originally noted by [17], *D. litoreus* and *D. halophilus* show a different morphology compared to all other *Dicranocentrus* species, with enlarged **a1**–**2** chaetae on the distal labrum, a sinuous ciliate mac on the proximal dens, mucronal spine absent and a reduced dorsal head and trunk macrochaetotaxy. Mari-Mutt compared the dorsal chaetotaxy of his two species to those of *Heteromurus*, in lacking the dorsal head **S0** chaeta, Th. III with five main mac (four in *Heteromurus*) and Abd. I devoid of mac [17,37,38]. This dorsal chaetotaxy, combined with antennae with five segments (only Ant. I subdivided) and mucro without spines, is more similar to the morphology of *Heteromurus s. str.* than to *Dicranocentrus*, although the annulated Ant. III is more similar to *Dicranocentrus* [8,33].

After our morphological study of the three previously described and the two new species of *Falcomurus* presented here, several shared features were noted between them and used to revise and complement the original diagnosis of the genus, especially regarding the main dorsal macrochaetotaxy. This character is mostly stable among the species, with few variations on the anterior and posterior head, Th. II **m4** complex and **p** series, plus central Abd. IV. Further characteristics may be shared among *Falcomurus* species, such as the presence of only two enlarged distal mac on the anterior face of the ventral tube [15] (p. 81, Figure 11, and the remarks we present on *F. chilikaensis*) and elements of the lateral chaetotaxy of the posterior head and trunk segments, which were omitted in [15,17]. On the other hand, the Ant. II undivided described for *F. chilikaensis*, *D. litoreus* and *D. halophilus* is not stable in the genus, and the two new species described here have the Ant. II subdivided, as seen in *Dicranocentrus*, *Pseudodicranocentrus* and *Sinodicranocentrus* [8].

A detailed comparison among the species is presented in *F. hilli* sp. nov. remarks. Further notes on the morphology of *Falcomurus* are presented in the first discussion topic.

### 3.2. Falcomurus chilikaensis Mandal, 2018

**Figure 2:**
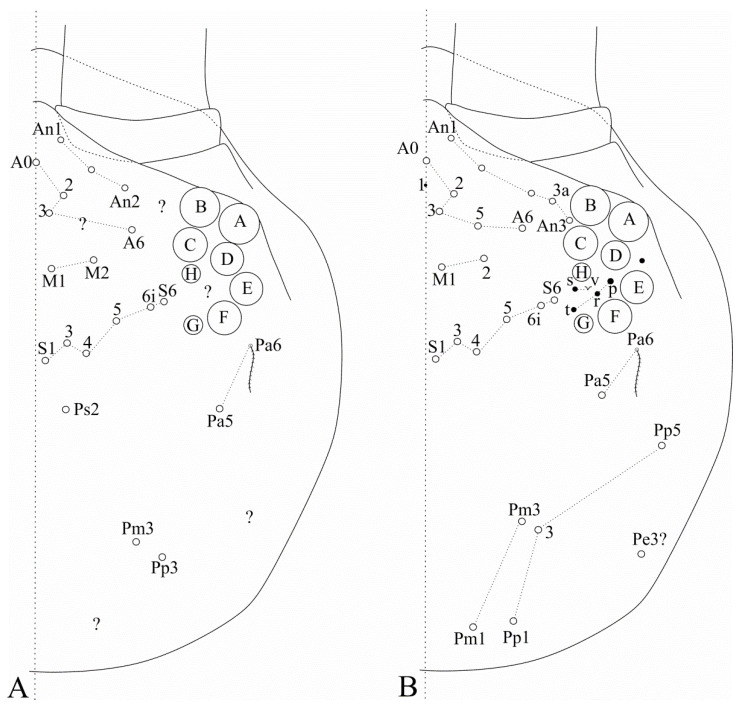
Dorsal head macrochaetotaxy, eyes and interocular chaetotaxy of (**A**) *Falcomurus chilikaensis*, where question marks represent unknown/unclear fields; (**B**) *Falcomurus halophilus* comb. nov., with the interocular **v** scale also as a mic.

**Figure 3:**
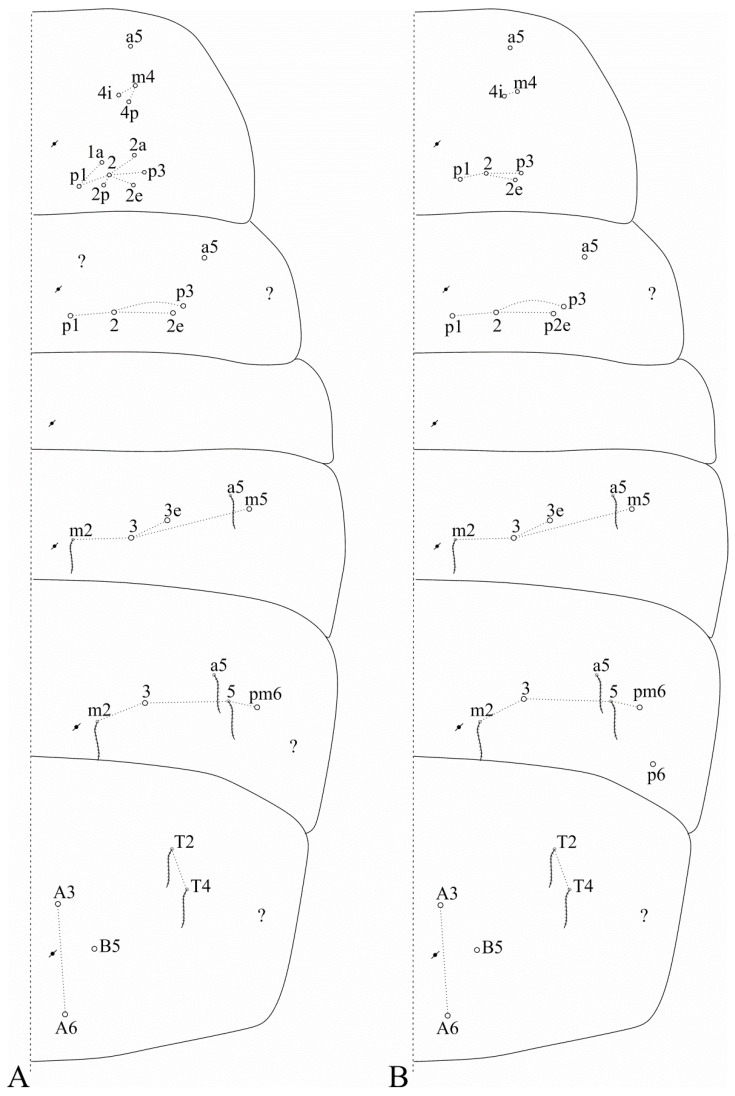
Dorsal trunk macrochaetotaxy of (**A**) *Falcomurus chilikaensis*; (**B**) *Falcomurus halophilus* comb. nov. Question marks represent unknown/unclear fields.

Figure 2A and Figure 3A.

*Falcomurus chilikaensis* Mandal, 2018 [15] (pp. 77–83, Figures 1–17), India, Odisha state, Ganjam district, Rambha town, nearby Sabulia village (original description).

Diagnosis: Dark blue/violet pigments over antennae, dorso-anterior head and tibiotarsi, light blue/violet pigments on femurs and Abd. VI. Ant. IV with a pin projection. Ant. II undivided. Labral **a2** chaeta normal, not enlarged as **a1**. Dorsal head with **Ps2** mac (Figure 2A); post-labial quadrangle with smooth chaetae. Th. II with three **m4** and seven **p** mac, respectively (Figure 3A). Abd. IV with 3 central mac (possibly **A3**, **A5** and **B5**) (Figure 3A). Trochanteral organ with 16–18 chaetae. Tibiotarsi proximally scaled. Unguiculi lacking the outer tooth. Dorsal manubrium with short smooth chaetae (adapted from [15]).

Remarks: It is quite probable that Mandal’s description of *F. chilikaensis* includes misinterpretations and omissions regarding the dorsal chaetotaxy and other morphological aspects, which we were able to track and correct after reviewing the other species. In this sense, it is worth noting we did not examine the *F. chilikaensis* type series.

For the dorsal head macrochaetotaxy [15] (p. 79, Figure 6), the common group of **A0**, **A2**–**3**, **A5** mac seen in several Heteromurinae and all other *Falcomurus* species were not clearly represented, and the marked **A0** actually belongs to the antennal series. So we considered in the anterior region the antennal series with three mac (originally represented only by one side), but quite possibly with more chaetae near the eyes, as we observed in the other taxa, and the original three anterior chaetae as misinterpretations of **A0**, **A2**–**3**, while **M3** is actually **A6**. Regarding the **M** and **S** series, the original drawing is not entirely symmetrical, so it is not clear if there are polymorphic chaetae, absent as mac in one of the sides. Since the author omitted mac on both sides in this drawing, we considered the absent mac of these series only as omissions and not as polymorphic chaetae, and such series were entirely represented in the left side of the original drawing. Concerning the posterior head, we believe the most posterior region was disregarded by the author, possibly due to the absence or reduction of mac, and the original drawing represents **Pa5**, almost universal to Entomobryoidea, **Pm3** and **Pp3**, seen in all other *Falcomurus* species, plus **Ps2**, exclusive of *F. chilikaensis*. It is also quite possible that *F. chilikaensis* has interocular chaetae, such as the other species and most, if not all, Entomobryoidea; but, they were overseen due to the presence of dark pigments over the eyepatches [15] (p. 79, Figure 3). On the labrum, Mandal’s representation of **a1** chaeta clearly marks it as enlarged on the left side, as described by [17] for *D. litoreus* and *D. halophilus*, while **a2** is apparently similar to the others [15] (p. 80, Figure 8).

For the dorsal trunk chaetotaxy [15] (p. 80, Figure 7), we considered the Th. II main chaetotaxy as it was presented by the author, since it fits the **a** and **m** series of the other species (but with **m4p**), while the **p** series shows some secondary multiples of **p1**–**2** mac, differently from other *Falcomurus* taxa but as seen in part of *Dicranocentrus* and some other Orchesellidae [6,7,8,29,32,33]. However, to Th. III until Abd. IV, Mandal’s representation of the dorsal mac encompasses chaetae from the left and right sides at the same time, being more complete on the right side. On the Th. III there are six posterior mac represented on the original drawing, but they are likely the left and right sets of **p1**–**2e**, seen in all other species of the genus. So, the two superior mac on the right side represent **p3** and **a5**, also seen in all other species of *Falcomurus*. The upper **a5** mac marked by the author could represent the **a1** mac seen in scaleless Orchesellidae, like *Orchesella* [32] (p. 152). However, since the **a1** mic in the new species occurs near to the pseudopore, next to **m1**, a feature seen in several other Heteromurini [3,8,27,33,34], we disregarded Mandal’s representation of this mac, considering it as a doubtful feature. On the Abd. II, Mandal’s description shows 3 bothriotricha; however, the Entomobryoidea present 2 + 2 bothriotricha on this segment [1,3,32]. In this segment, the author actually represents, from the left to the right: the left **m3** mac, left **m2** bothriotrichum, right **m2** bothriotrichum, right **m3**–**3e** mac, right **a5** bothriotrichum and right **m5** mac, all also seen in the other species of the genus. A similar mistake was seen in the Abd. III, which originally represents, from the left to the right: the left **m3** mac (left **m2** bothriotrichum was overseen, but its alveolus is smaller than the mac ones), right **m2** bothriotrichum, right **m3** mac, right **a5** and **m5** bothriotricha and right **pm6** mac, similar to its congeners. In this segment, it is quite possible that the **p6** mac is also present, and it was not represented since it did not fit the drawing. The Abd. IV interpretation was quite puzzling, since the original drawing was different from the expected disposition of *Dicranocentrus* and *Heteromurus* central mac [29,33,37]. *Falcomurus halophilus* comb. nov. also has three central mac on Abd. IV, **A3**, **A6** and **B5**, while the other species of the genus have **A6** as a mes, so we believe the original drawing of *F. chilikaensis* shows the chaetae inverted, and they are actually the same three. Once again, the left side is incomplete, as for the most trunk segments, so we disregarded a possible polymorphic state of **A6** and considered the Abd. IV with three internal mac.

Regarding other characteristics, in Ant. IV we disregarded the presence of the antennal bulb, as noted by [8], but considered the left side structure as a pin projection (see [15] (p. 79, Figure 5) and [27] (p. 4, Figure 2B)). Mandal’s description noted his species had all legs scaled; however, the author did not remark on the presence or absence of scales on any particular segment of the legs. In his depiction of the tibiotarsi, the superior (proximal) region has “spine-like” structures, which most likely represent the scales alveoli, as they are somewhat pointed [15] (p. 81, Figure 12). The habitus photograph of *F. chilikaensis* also suggest such scales are present, as seen in the right fore and the left hind legs [15] (p. 78, Figure 1). In addition, all specimens of *Falcomurus* we revised do not have short spines on the tibiotarsi. Because of this, we considered the tibiotarsi of *F. chilikaensis* proximally scaled, as we observed in *F. litoreus* comb. nov. and *F. hilli* sp. nov. Finally, the drawing of the ventral tube corpus in Mandal’s description [15] (p. 81, Figure 11) possibly represents the lateral region, which is devoid of chaetae in many Entomobryoidea and also in other *Falcomurus* species. So, the right side of the drawing possibly represents the two distal macrochaetae (as in [17] (p. 314, Figure 78)) and the left side, one of the outer posterior ciliate chaetae.

After our reinterpretation of the original *F. chilikaensis* description, we believe the type material should be re-examined and the taxon redescribed, to confirm its morphology is similar to other Heteromurinae taxa as herein discussed. In this sense, our diagnosis, notes and drawings of this species should be taken as provisional, until further data based on the type specimens are published.

### 3.3. Falcomurus halophilus (Mari-Mutt, 1985) comb. nov.

Figure 2B and Figure 3B.

*Dicranocentrus halophilus* Mari-Mutt, 1985 [17] (pp. 315, 320, Figures 79–82), Papua New Guinea, Morobe, Lae (original description).

Diagnosis: Dark blue/violet pigments over all antennal segments, tibiotarsi, dorso-anterior head and around the mouth, light blue/violet uniformly distributed over trunk, other leg segments, ventral tube corpus and manubrium. Ant. IV without pin projection. Ant. II undivided, without scales. Labral **a1**–**2** chaetae enlarged, distal labrum with two distinct pointed papillae. Dorsal head with **Pm1**, **Pp1**, **Pp5** and **Pe3?** mac, without **An1a** and **Ps2** mac, interocular area with five ciliate chaetae or four ciliate chaetae and one scale (Figure 2B). Mandible apex curved to the midline. Post-labial quadrangle without smooth chaetae, anterior post-labial region with a pair of smooth chaetae near the labium. Th. II with two **m4** and four **p** mac, respectively (Figure 3B). Abd. IV with three central mac (**A3**, **A6** and **B5**) (Figure 3B). Unguiculi with the outer tooth. Dorsal manubrium without smooth chaetae (adapted from [17]).

Remarks: *Dicranocentrus halophilus* is herein transferred to *Falcomurus* since it shares characteristics with other species of the genus, especially the absence of **A1**, **M3**–**4**, **S0** and **S2** mac on dorsal head, the overall reduction of main dorsal macrochaetotaxy on trunk, including the Abd. I lacking mac, ungues short and thick, internally with only the basal paired teeth, unguiculi excavated, dens lacking spines, proximally with a falcate ciliate mac, and mucro lacking the mucronal spine.

Our diagnosis of *F. halophilus* comb. nov. was based on the original description, since we did not revise the type specimens. As Mari-Mutt’s description of this species noted mostly its dissimilarities with *F. litoreus* comb. nov., we used some data of the latter species to complement the diagnosis of *F. halophilus* comb. nov. [17] (pp. 319–320). On the other hand, the exact number of trochanteral spines and the presence of scales on the tibiotarsi and ventral tube anterior face, seen in our revision of *F. litoreus* comb. nov. (see the next species), were not considered for *F. halophilus* comb. nov., as they were not mentioned by the author. Concerning the interocular chaetotaxy of this species, the author described it with one outer and three inner chaetae plus a scale, similarly to *F. litoreus* comb. nov. However, in his drawing [17] (p. 315, Figure 80), there are only three interocular chaetae and the scale. We believe both Mari-Mutt species have a similar interocular chaetotaxy. Even so, the author regarded the interocular scale as a chaeta in one specimen [17] (p. 320).

### 3.4. Falcomurus litoreus (Mari-Mutt, 1985) comb. nov.

**Figure 4:**
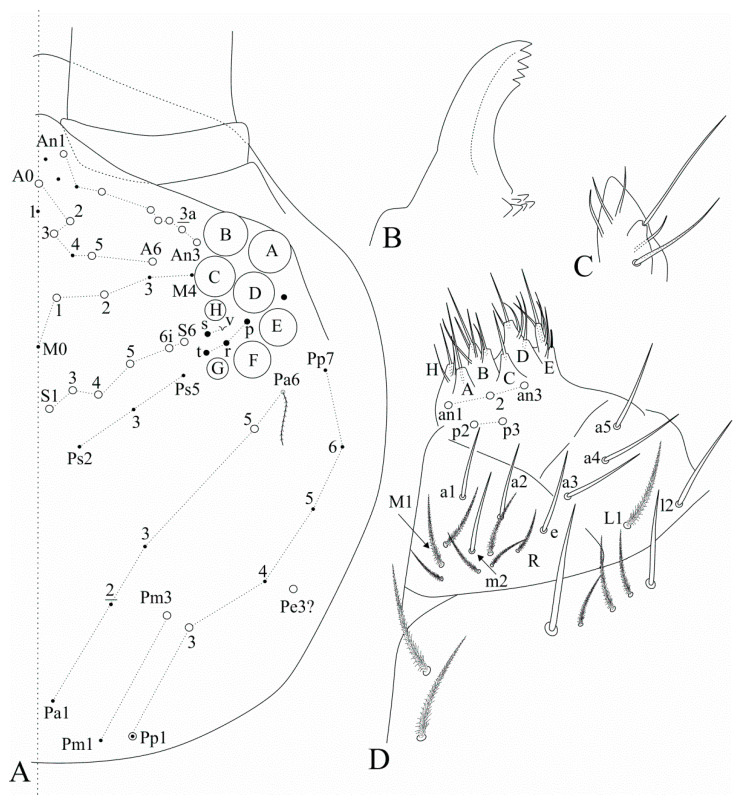
*Falcomurus litoreus* comb. nov. head: (**A**) dorsal head chaetotaxy and eyes, right side, interocular **s** chaeta also as a scale; (**B**) left mandible apex; (**C**) right maxillary outer lobe and sublobal plate; (**D**) right labium and anterior region of post-labial field, including the post-labial quadrangle chaetae.

**Figure 5:**
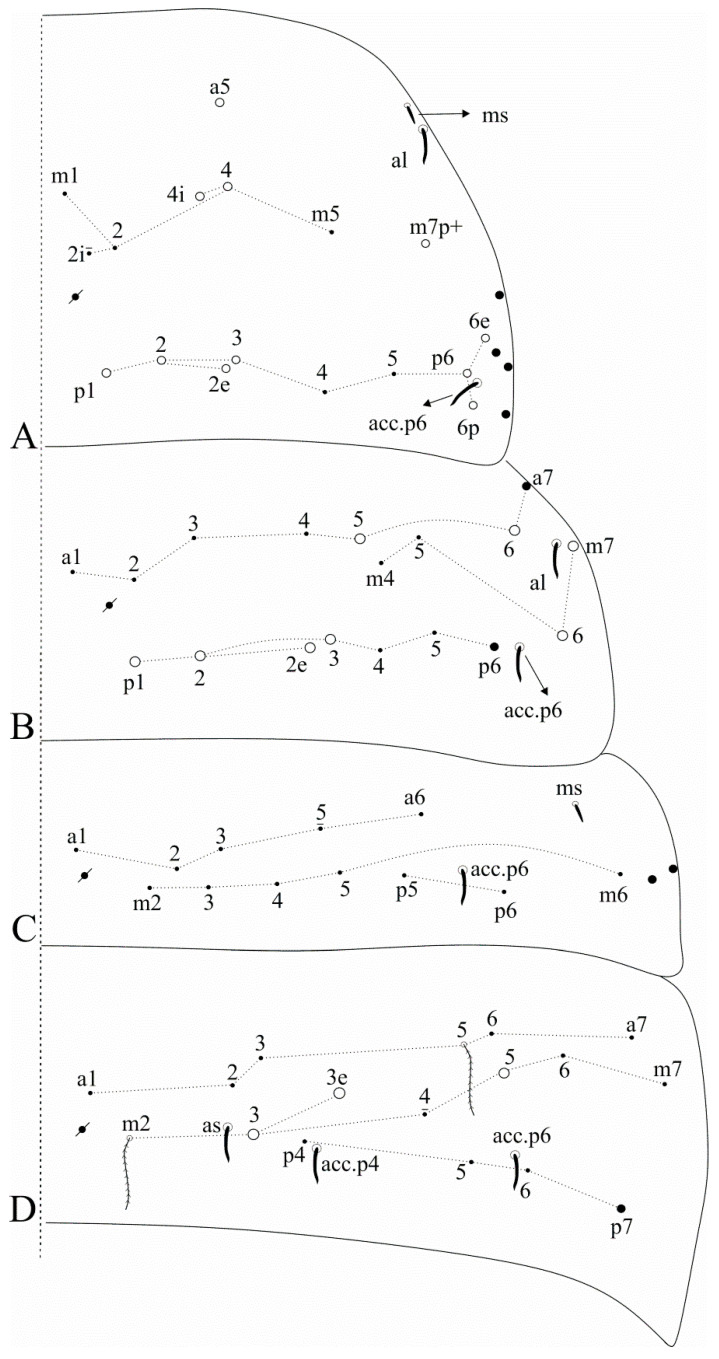
*Falcomurus litoreus* comb. nov. trunk dorsal chaetotaxy, right side: (**A**) Th. II; (**B**) Th. III; (**C**) Abd. I; (**D**) Abd. II.

**Figure 6:**
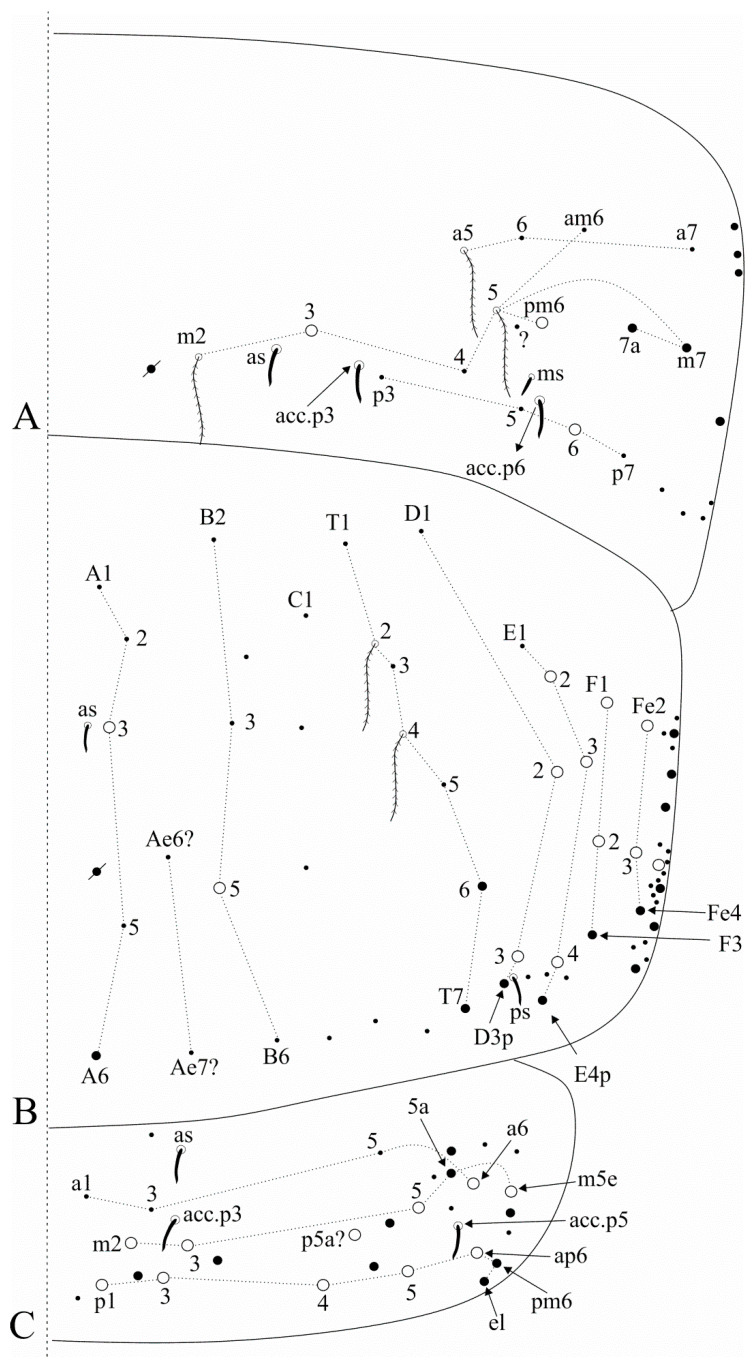
*Falcomurus litoreus* comb. nov. trunk dorsal chaetotaxy, right side: (**A**) Abd. III; (**B**) Abd. IV; (**C**) Abd. V (main chaetotaxy).

**Figure 7:**
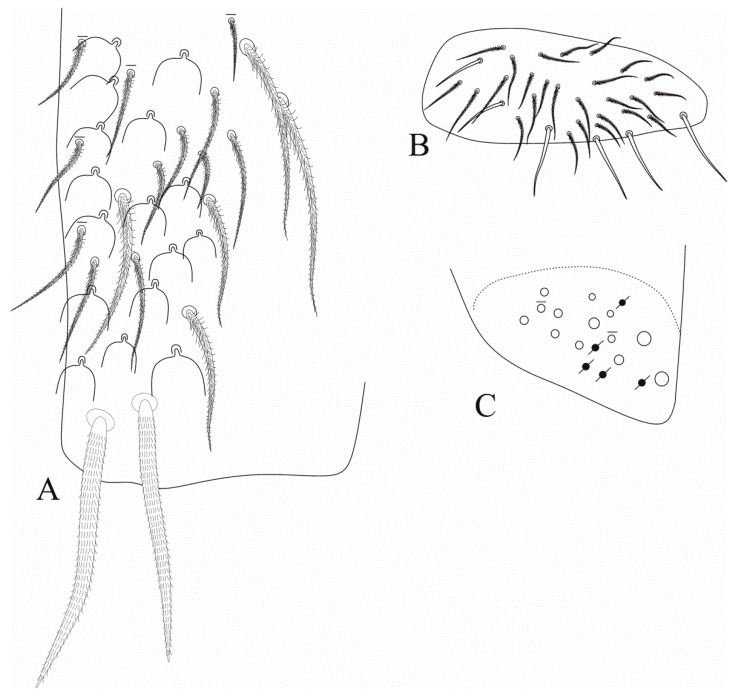
*Falcomurus litoreus* comb. nov. abdominal appendages: (**A**) ventral tube anterior face, right side; (**B**) lateral flap, right side; (**C**) left manubrial plate.

Figure 4, Figure 5, Figure 6 and Figure 7.

*Dicranocentrus litoreus* Mari-Mutt, 1985 [17] (pp. 313–314, 318–320, Figures 64–78, The Philippines, Mindoro, Puerto Galera (original description).

Examined material: Holotype (sex unknown) and 4 paratypes (one female, others unknown). The Philippines, Mindoro, Puerto Galera, on the beach, 27–29.xii.1979. All deposited now at MVMA.

Diagnosis: Dark blue/violet pigments over distal Ant. Ib, II and entire III (IV unknown), dorso-anterior head and tibiotarsi, light blue/violet pigments on Th. I, coxae, trochanters and distal ventral tube corpus; trunk devoid of pigments or brownish, with a median unpigmented line from Th. II to Abd. III. Ant. II undivided, with scales. Labral **a1**–**2** chaetae enlarged, **m2** normal, two distal labrum papillae pointed but weakly developed. Dorsal head with **Pp1** (also as mic) and **Pe3?** mac, without **An1a**, **Ps2**, **Pm1** and **Pp5** mac, interocular area with four ciliate chaetae and one scale or three ciliate chaetae and two scales. Mandible apex curved to the midline. Post-labial quadrangle without smooth chaetae, anterior post-labial region with a pair of smooth chaetae near the labium. Th. II with two **m4** and four central **p** mac, respectively. Abd. IV with two central mac (**A3** and **B5**). Ventral tube densely scaled, lateral flap with about six smooth and 26 ciliate chaetae. Trochanteral organ with 27–32 chaetae. Tibiotarsi proximally scaled. Unguiculi with the outer tooth. Dorsal manubrium without short smooth chaetae, manubrial plate with 11–13 chaetae.

Additions to the original description: Ventral head and anterior face of the ventral tube densely covered by scales, tibiotarsi scaled proximally.

Head (Figure 4). Two inner pointed labral papillae present, but underdeveloped. Interocular field with four mes (**s**, **t**, **r** and **p**) plus **v** as a scale, **s** also as a scale in one side of one specimen. Head dorsal macrochaetotaxy antennal (**An**) row with 6–7 mac, including **An3**, **An1a** absent, anterior (**A**) row with five (**A0**, **A2**–**3**, **A5**–**6**), medio-ocellar (**M**) row with two (**M1**–**2**), **M0** mic present; sutural (**S**) row with six (**S1**, **S3**–**6**), post-occipital anterior (**Pa**) row with one (**Pa5**), post-occipital medial with one (**Pm3**), post-occipital posterior (**Pp**) with 1–2 (**Pp1** and **Pp3**, **Pp1** also as mic) and post-occipital external with one (**Pe3?**) mac; further details are represented in Figure 4A. Mandible apex somewhat curved to the midline (Figure 4B). Maxillary outer lobe basal chaeta acuminate, slender (not erect or spiniform) and rough, shorter than the apical one, sublobal plate with four chaeta-like appendages, subequal in size (Figure 4C). Labium with six main papillae (**H** plus **A**–**E**), with 2, 0, 5, 0, 4, 4 guard chaetae, respectively, with five proximal chaetae subequal in length (**an1**–**3**, **p2**–**3**); labium unscaled, labial basomedial (submentum or labial triangle) and basolateral (mentum) fields with chaetae **a1**–**5**, **m2**, **e** and **l2** smooth, **l2** also ciliate ([17], p. 319), basomedian field with 5–7 ciliate chaetae (analysed specimen with seven), including **M1** and **R**, basolateral **L1** ciliate, subequal in length to **l2**. Post-labial region with several scales and ciliate chaetae, except for the two smooth anterior chaetae nearby the labium, post-labial quadrangle with two subequal ciliate chaetae (Figure 4D).

Trunk dorsal chaetotaxy (Figure 5 and Figure 6). Tergal S-chaetotaxy from Th. II to Abd. V as 2,2|1,3,3,+,3 for sens, and 1,0|1,0,1,0,0 for ms. Th. II, excluding the anterior collar, with one anterior (**a5**), three medial (**m4**–**4i** and a multiple of **m7p** row far from the collar) and seven posterior (**p1**–**3**, **p6**–**6e**) mac (Figure 5A). Th. III with two anterior (**a5**–**6**), two medial (**m6**–**7**) and four posterior (**p1**–**3**) mac, **m1** mic not seen but possibly present (Figure 5B). Abd. I lacking mac, **p5** mic present (Figure 5C). Abd. II with three medial (**m3**–**3e**, **m5**) mac (Figure 5D). Abd. III with two medial (**m3** and **pm6**) and one posterior (**p6**) mac, one mic without clear homology near **pm6**, **a1**–**3** and **m3e** mic not seen, but at least **a1**–**3** possible present, lateral tergum with at least eight extra chaetae (Figure 6A). Abd. IV with two central (**A3** and **B5**) and 10 lateral (**D2**–**3**, **E2**–**4**, **F1**–**2**, **Fe2**–**3**, plus an external one) mac, **as** sens internal to **A3**, **ps** nearby **D3p**, **Ae6**–**7** only seen by alveoli, lateral tergum with at least 17 mic and six mes without clear homologies (Figure 6B). Abd. V main chaetotaxy with one anterior (**a6**), four medial (**m2**–**3**, **m5**, **m5e**), five posterior (**p1**, **p3**–**ap6**) plus **p5a?** mac, with several secondary chaetae (only few represented in the drawing), sensillar pattern similar to *Heteromurus* (as in [3,8]) (Figure 6C). More details on the trunk’s idio and S-chaetotaxy are represented in Figure 5 and Figure 6.

Trunk appendages (Figure 7). Ungues lateral paired teeth present and underdeveloped. Ventral tube anteriorly without smooth chaetae, with scales from the basis to the apex, with 12–17 ciliate chaetae of different sizes plus the distal two mac (Figure 7A). Posterior face densely covered by ciliate chaetae and some central smooth chaetae, not entirely clear in the analysed specimens. Lateral flaps with about six smooth and 26 ciliate chaetae each, four distal smooth chaetae larger than the others (Figure 7B). Manubrial plate with 11–13 ciliate chaetae and five small pseudopores (Figure 7C).

Remarks: *Dicranocentrus litoreus* is herein transferred to *Falcomurus* as it shares with other species mainly the following combination of characters: absence of **A1**, **M3**–**4**, **S0** and **S2** mac on the dorsal head; the reduction of the main dorsal macrochaetotaxy on the trunk, as described in the genus diagnosis; a foot complex with a short unguis holding internally only the proximal paired teeth; unguiculi excavated; dens devoid of spines, with the typical proximal falcate mac seen in the genus; and the mucronal spine absent.

Our revision of *F. litoreus* comb. nov. type material confirmed most of the data provided by [17], with few corrections: the antennal series could present one more mac and the posterior head could also present **Pp1** as mac (seen only in one side of one specimen), while the ventral tube anterior region does not hold smooth chaetae [17] (p. 314, Figure 78). We also could confirm the Ant. II undivided and the interocular element **v** as a scale, as noted by the author [17] (p. 318). We disagree with the original description of the mandible apex, described as elongated. In this case, it is quite similar to *F. pulukokos* sp. nov. in length, but it is somewhat curved internally.

Unfortunately, we could only revise the dorsal chaetotaxy of the trunk in one specimen, which was damaged near the midline, so we could not confirm the presence of some internal primary mic nor identify further polymorphic chaetae. Due to the poor preservation of all five analysed slides, we could not properly revise the antennae, ventral tube posterior face and ventro-distal manubrium.

### 3.5. Falcomurus pulukokos sp. nov. Bellini, Souza and Greenslade

**Figure 8:**
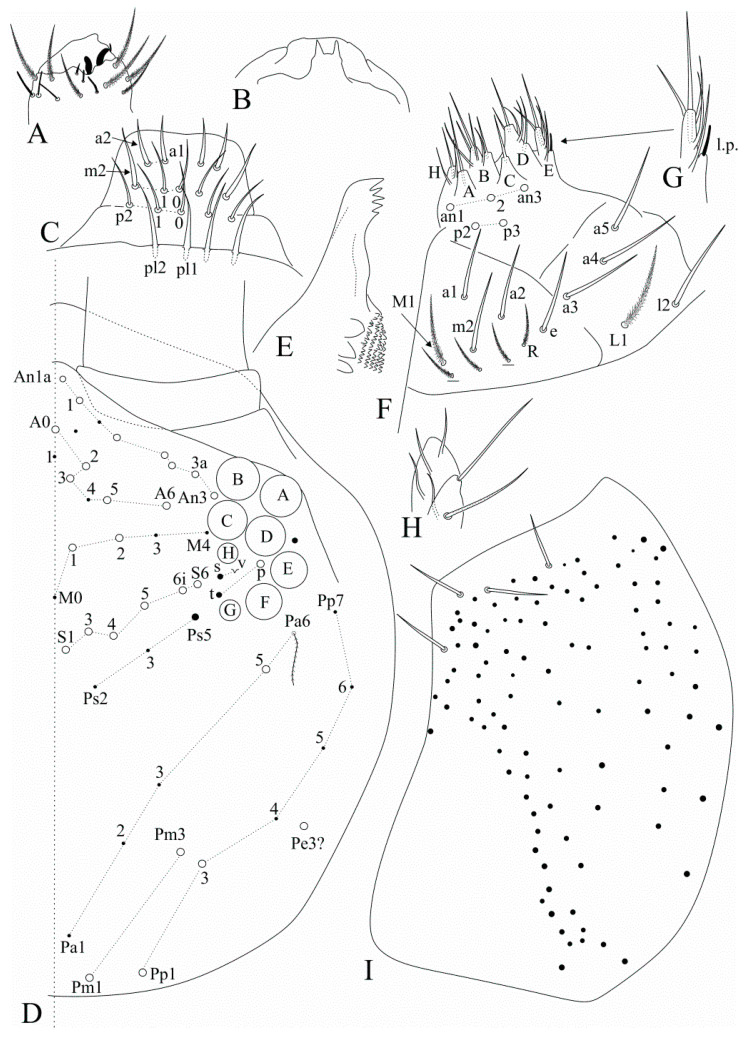
*Falcomurus pulukokos* sp. nov. head: (**A**) ventro-apical region of Ant. III, left antenna; (**B**) labral papillae; (**C**) labral and prelabral chaetotaxy; (**D**) dorsal head chaetotaxy and eyes, right side; (**E**) left mandible apex; (**F**) right labium; (**G**) labial papilla **E** in detail; (**H**) right maxillary outer lobe and sublobal plate; (**I**) post-labial chaetotaxy, right side, scales omitted.

**Figure 9:**
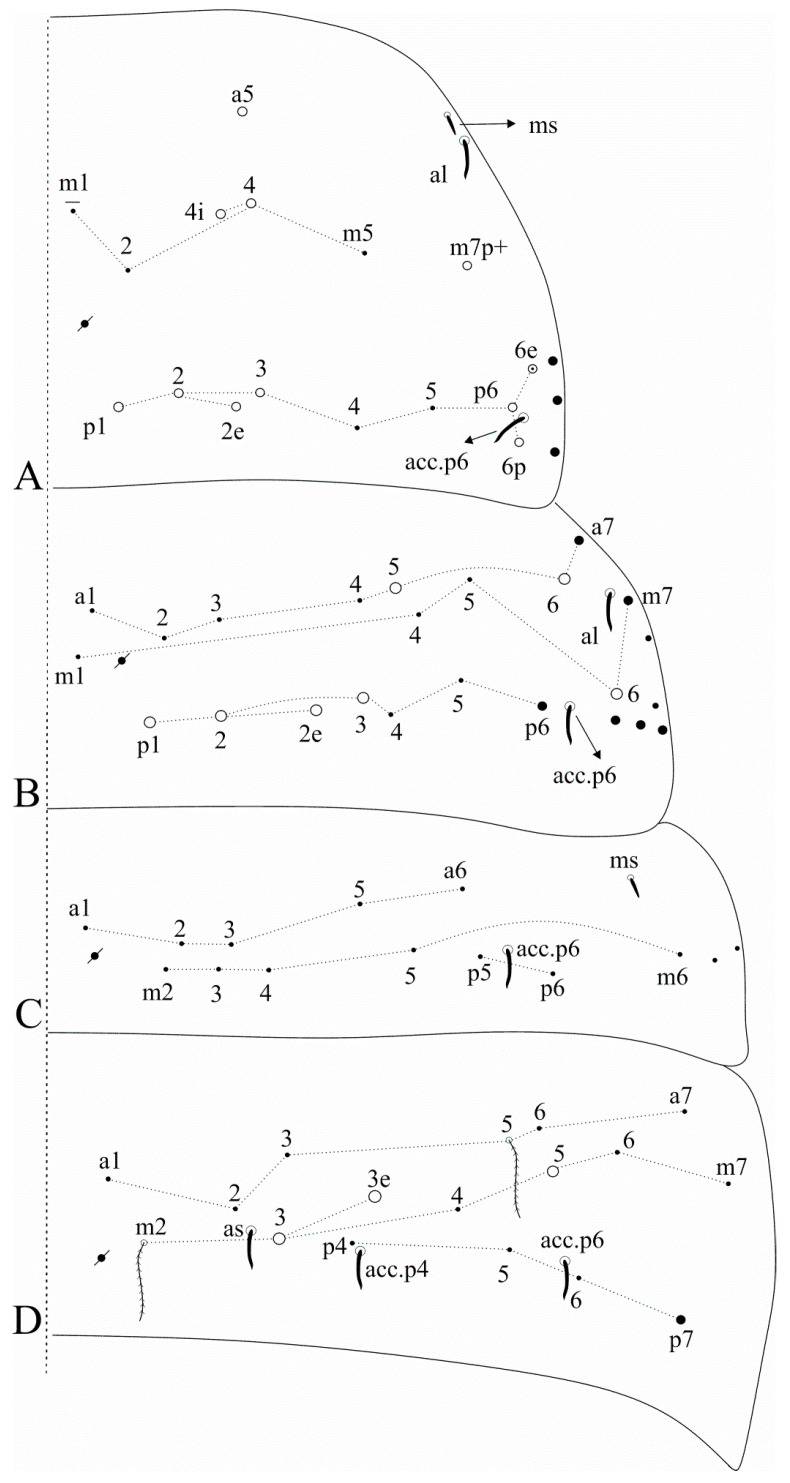
*Falcomurus pulukokos* sp. nov. trunk dorsal chaetotaxy, right side: (**A**) Th. II; (**B**) Th. III; (**C**) Abd. I; (**D**) Abd. II.

**Figure 10:**
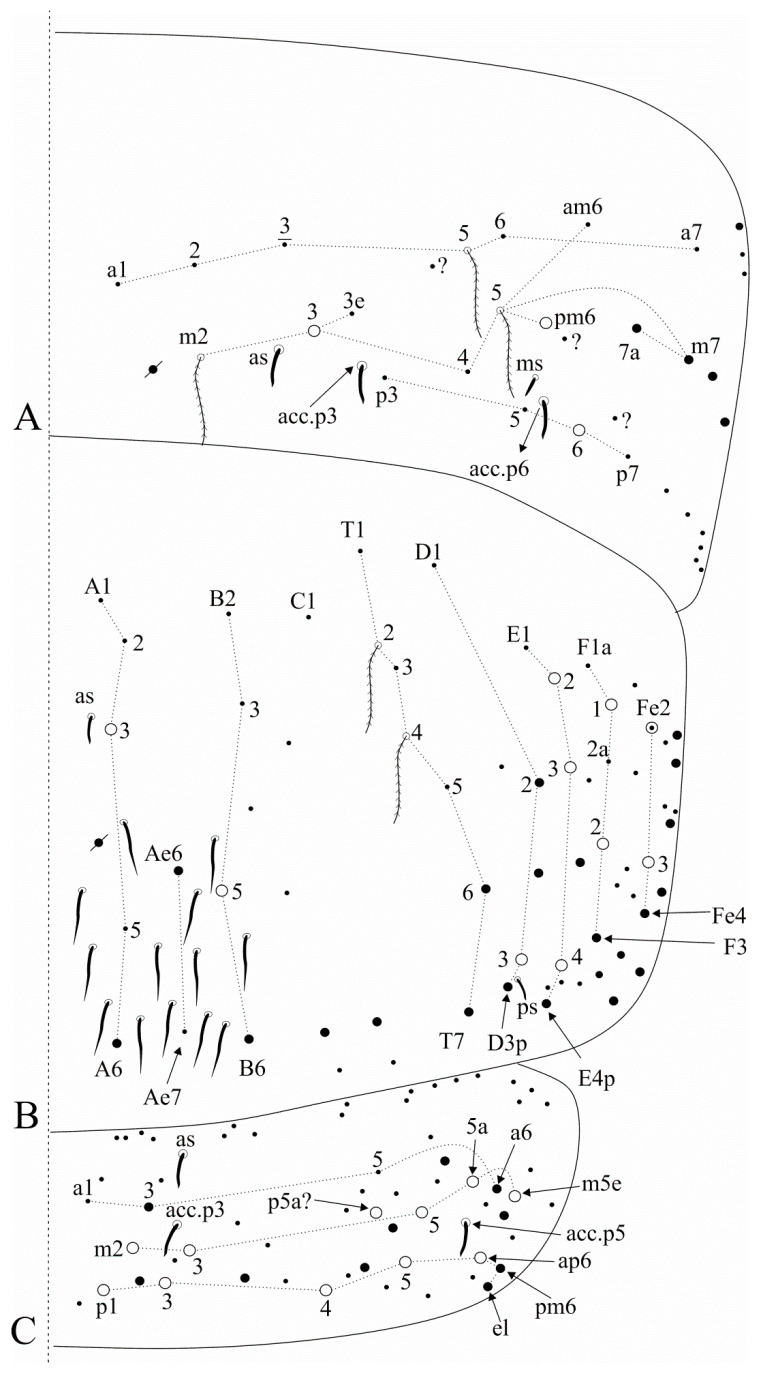
*Falcomurus pulukokos* sp. nov. trunk dorsal chaetotaxy, right side: (**A**) Abd. III; (**B**) Abd. IV; (**C**) Abd. V.

**Figure 11:**
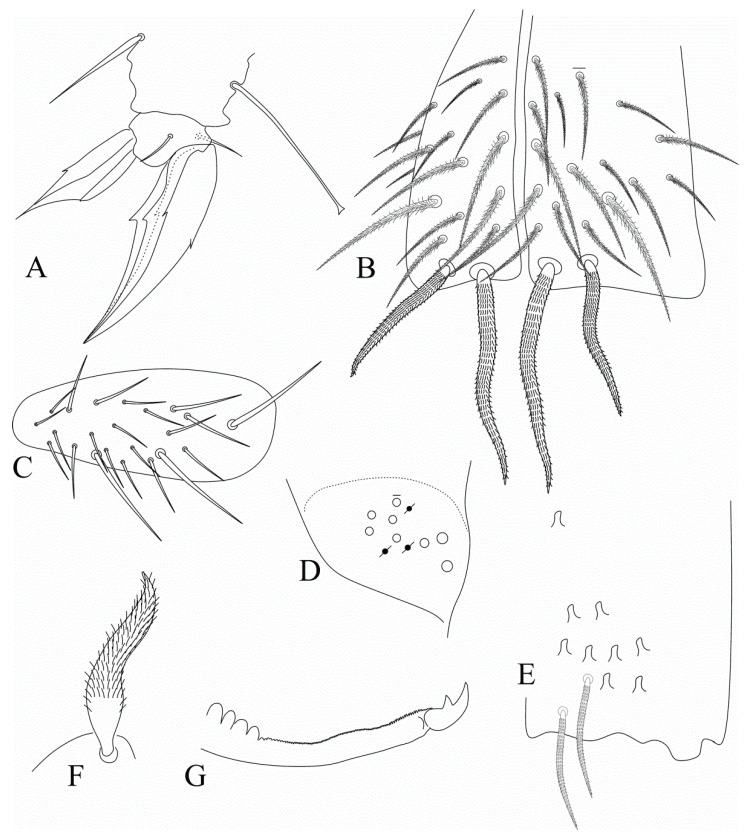
*Falcomurus pulukokos* sp. nov. trunk appendages: (**A**) foot complex of leg III; (**B**) ventral tube anterior face; (**C**) lateral flap, left side; (**D**) left manubrial plate; (**E**) ventro-distal manubrium, right side; (**F**) sinous mac on dorso-proximal dens; (**G**) distal dens and mucro, chaetae omitted.

Figure 8, Figure 9, Figure 10 and Figure 11.

Type material: Holotype: female in slide (MVMA), along with two specimens of *Dicranocentrus* sp.: Australia, Cocos and Keeling Islands, West Island (12°09′ S, 96°49′ E), v–vi.2005, leaf litter light trap near the beach, A. Yen coll. Paratype in slide (MVMA): 1 female, along with two specimens of *Dicranocentrus* sp., same data of holotype.

Diagnosis: Ant. IV without pin projection, Ant. II subdivided in IIa and IIb, with few scales. Labral **a1**–**2**, **m2** chaetae enlarged, two inner labral papillae present and well developed. Dorsal head with **An1a**, **Pm1**, **Pp1** and **Pe3?** mac, without **Ps2** and **Pp5** mac, interocular area with three ciliate chaetae and one scale. Mandible apex normal, not elongated, narrow or curved. Post-labial quadrangle with two smooth chaetae, anterior post-labial region with a pair of smooth chaetae near the labium. Th. II with two **m4** and four central **p** mac, respectively. Abd. IV with two central mac (**A3** and **B5**). Ventral tube without scales, lateral flap with about 22 smooth chaetae. Trochanteral organ with at least 20 chaetae. Tibiotarsi unscaled. Unguiculi with the outer tooth. Dorsal manubrium without short smooth chaetae, manubrial plate with 7–8 chaetae.

Description: Body length (head + trunk) of holotype = 1.36 mm; range of type series length = 1.28–1.36 mm; average body length = 1.32 mm. Colour pattern unknown. Coarsely ciliate apically rounded or truncate scales present on Ant. I–II, head (dorsally and ventrally), dorsal trunk, legs (with exception of tibiotarsi), ventral and lateral manubrium and dens. Ventral tube unscaled.

Head (Figure 8). Antennae shorter than body, ratio antennae:body of holotype = 1:1.84; Ant. III slightly smaller than Ant. IV, antennal ratio Ant. Ia–IV of holotype = 1:7:1.6:9.6:20:21. Ant. IV and III typically annulated. Ant. IV without a subapical organite, pin projection or any clear differentiated apical sens, only with normal sens and ciliate chaetae. Ant. III sense organ with two swollen sensory rods, three guard sensilla (all nearby the sensory rods) plus at least four surrounding regular sens (Figure 8A). Ant. II subdivided in IIa and IIb, IIb with few scales, ventral face not entirely clear, with a single long smooth chaeta on the upper central region, dorsally with two apical sensory rods. Two inner pointed labral papillae present and well developed (Figure 8B). Prelabral chaetae smooth and longer than the labral ones, labral **a1**–**2** and **m2** enlarged, **m2** slightly longer than other labral chaetae (Figure 8C). Eyepatches lenses A–F subequal in size, G–H smaller than the others, with four interocular elements: **s**, **t** and **p** chaetae, the latter as a mac, and **v** as a scale. Head dorsal macrochaetotaxy antennal (**An**) row with seven, including **An1a**, anterior (**A**) row with five (**A0**, **A2**–**3**, **A5**–**6**), medio-ocellar (**M**) row with two (**M1**–**2**), **M0** mic present; sutural (**S**) row with six (**S1**, **S3**–**6**), post-occipital anterior (**Pa**) row with one (**Pa5**), post-occipital medial with two (**Pm1** and **Pm3**), post-occipital posterior (**Pp**) with two (**Pp1** and **Pp3**) and post-occipital external with one (**Pe3?**) mac; further details are represented in Figure 8D. Mandibles normal, not remarkably elongated, narrow or curved, with 4–5 apical incisive teeth (Figure 8E). Maxillae capitulum similar to *F. litoreus* comb. nov. [17] (p. 314, Figure 75). Labium with six main papillae (**H** plus **A**–**E**), with 2, 0, 5, 0, 4, 4 guard chaetae, respectively, papilla **E** lateral process finger-shaped and short, not reaching the papilla apex; with five proximal chaetae subequal in length (**an1**–**3**, **p2**–**3**); labium unscaled, labial basomedial (submentum or labial triangle) and basolateral (mentum) fields with chaetae **a1**–**5**, **m2**, **e** and **l2** smooth, basomedian field with 3–5 ciliate chaetae including **M1** and **R**, basolateral **L1** ciliate, subequal in length to **l2** (Figure 8F,G). Maxillary outer lobe basal chaeta acuminate, slender (not erect or spiniform) and rough, shorter than the apical one, ratio basal chaeta:apical chaeta of holotype = 1:1.15; sublobal plate with four chaeta-like appendages, subequal in size (Figure 8H). Post-labial chaetotaxy with four smooth chaetae, two on the post-labial quadrangle and two on the first post-labial anterior row, and about 97 ciliate chaetae with different sizes (Figure 8I).

Trunk dorsal chaetotaxy (Figure 9 and Figure 10). Tergal S-chaetotaxy from Th. II to Abd. V as 2,2|1,3,3,+,3 for sens, and 1,0|1,0,1,0,0 for ms. Th. II, excluding anterior collar, with one anterior (**a5**), three medial (**m4**–**4i** and a multiple of **m7p** row far from the collar) and 6–7 posterior (**p1**–**3**, **p6**–**6e**, **p6e** also as mic) mac (Figure 9A). Th. III with two anterior (**a5**–**6**), one medial (**m6**) and four posterior (**p1**–**3**) mac (Figure 9B). Abd. I lacking mac, **p5** mic present (Figure 9C). Abd. II with three medial (**m3**–**3e**, **m5**) mac (Figure 9D). Abd. III with two medial (**m3** and **pm6**) and one posterior (**p6**) mac, three mic without clear homologies, lateral tergum with at least 11 extra chaetae (Figure 10A). Abd. IV with two central (**A3** and **B5**) and 7–8 lateral (**D3**, **E2**–**4**, **F1**–**2** and **Fe2**–**3**, **Fe2** also as mic) mac, **as** sens internal to **A3**, **ps** nearby **D3p**, central region with at least 13 long sens (possibly many more) plus five mes (**A6**, **Ae6**, **B6** and two without clear homologies), lateral tergum with at least 12 mic and 10 mes without clear homologies (Figure 10B). Ratio Abd. III:IV of holotype 1:1.63. Abd. V with five medial (**m2**–**3**, **m5**–**5e**), five posterior (**p1**, **p3**–**ap6**) plus **p5a?** mac plus several secondary chaetae, sensillar pattern similar to *Heteromurus* (as in [3,8]) (Figure 10C). More details on the trunk’s idio and S-chaetotaxy are represented in Figure 9 and Figure 10.

Trunk appendages (Figure 11). Trochanteral organ unclear, with at least 20 spine-like smooth chaetae. Femurs and tibiotarsi lacking extra smooth chaetae. Tenent-hairs capitate, slightly shorter than ungues; tibiotarsus III smooth inner distal chaeta present; anterior and posterior pretarsal chaetae present and well developed; ungues with a pair of lateral underdeveloped teeth on proximal 1/3, inner side with only the two basal paired teeth on proximal 1/3, internal lamellae only merging at the apex, dorsal tooth present, almost in the middle of the unguis; unguiculi excavated, all lamellae smooth, except for the postero-external with an external tooth in its distal half (Figure 11A). Ventral tube anteriorly without smooth chaetae, with two distal mac (the inner larger than the outer one), plus 15–16 ciliate chaetae of different sizes (Figure 11B). Posterior face unclear, but apparently with several lateral ciliate chaetae and few centro-distal smooth chaetae. Lateral flaps with about 22 smooth chaetae each, three of them larger than the others (Figure 11C). Tenaculum corpus with a single clearly ciliate chaeta, each ramus with four teeth. Manubrium and dens lacking smooth chaetae of any size. Manubrial plate with 7–8 chaetae, the two internal slightly larger than the others, plus at least three small pseudopores, possibly more (Figure 11D). Ventro-distal manubrium with two inner ciliate chaetae plus about eight scales (Figure 11E). Dorso-proximal dens with a typical sinuous ciliate mac (Figure 11F), mucro apical tooth larger than the basal one, mucronal spine typically absent (Figure 11G).

Etymology: The epithet refers to the Malay’s name for the Keeling territory.

Remarks: A detailed comparison of *Falcomurus* species is presented in the remarks of the next species.

### 3.6. Falcomurus hilli sp. nov. Bellini, Souza and Greenslade

**Figure 12:**
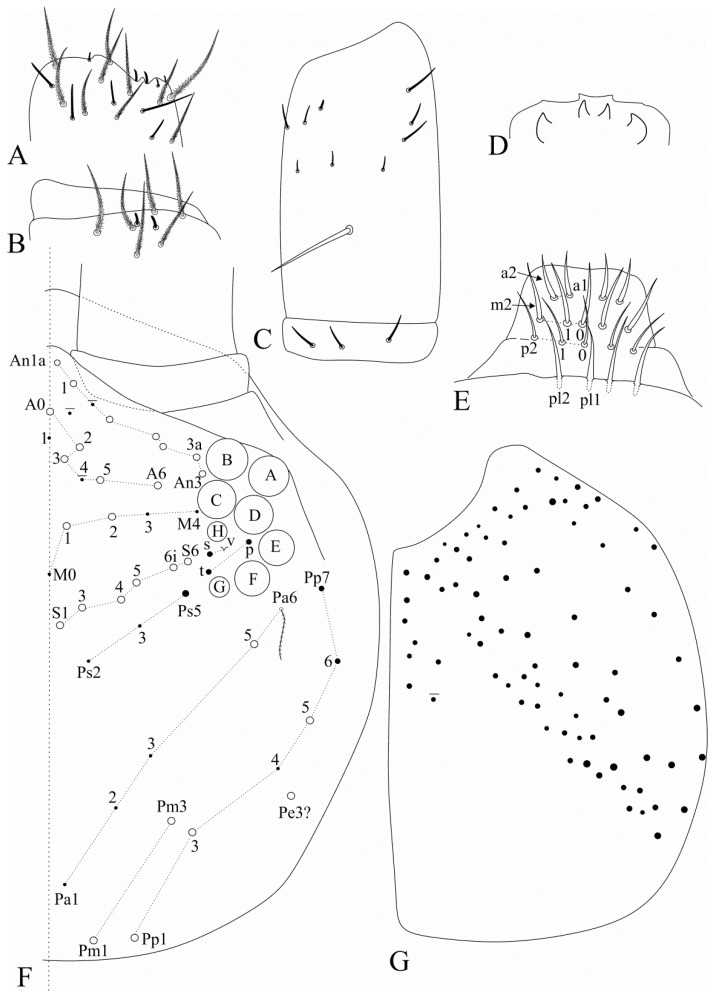
*Falcomurus hilli* sp. nov. head: (**A**) ventro-apical region of Ant. III, right antenna; (**B**) dorso-apical region of Ant. II, right antenna; (**C**) ventral Ant. II main sens and smooth chaeta, left antenna; (**D**) labral papillae; (**E**) labral and prelabral chaetotaxy; (**F**) dorsal head chaetotaxy and eyes, right side; (**G**) post-labial chaetotaxy, right side, scales omitted.

**Figure 13:**
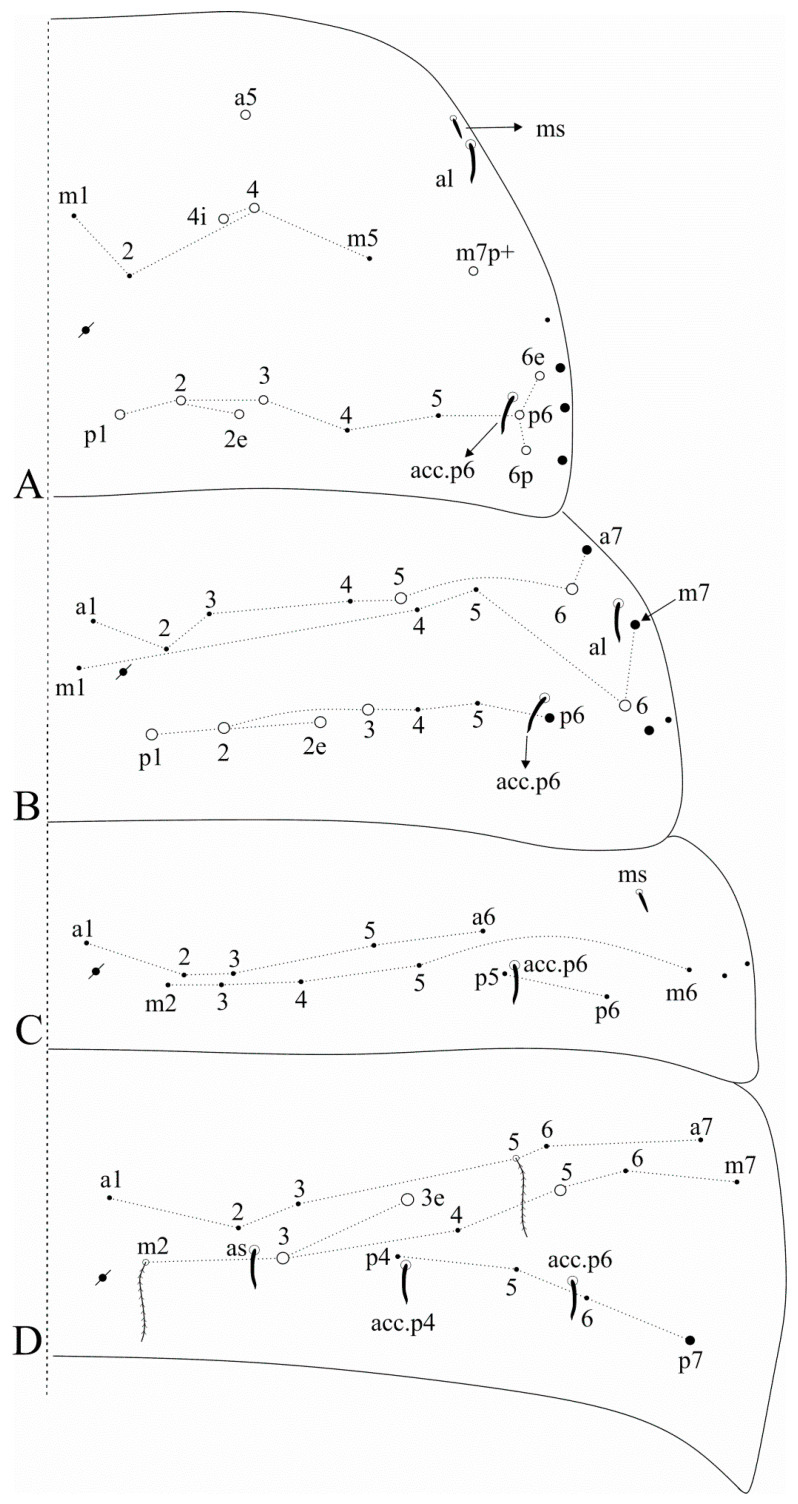
*Falcomurus hilli* sp. nov. trunk dorsal chaetotaxy, right side: (**A**) Th. II; (**B**) Th. III; (**C**) Abd. I; (**D**) Abd. II.

**Figure 14:**
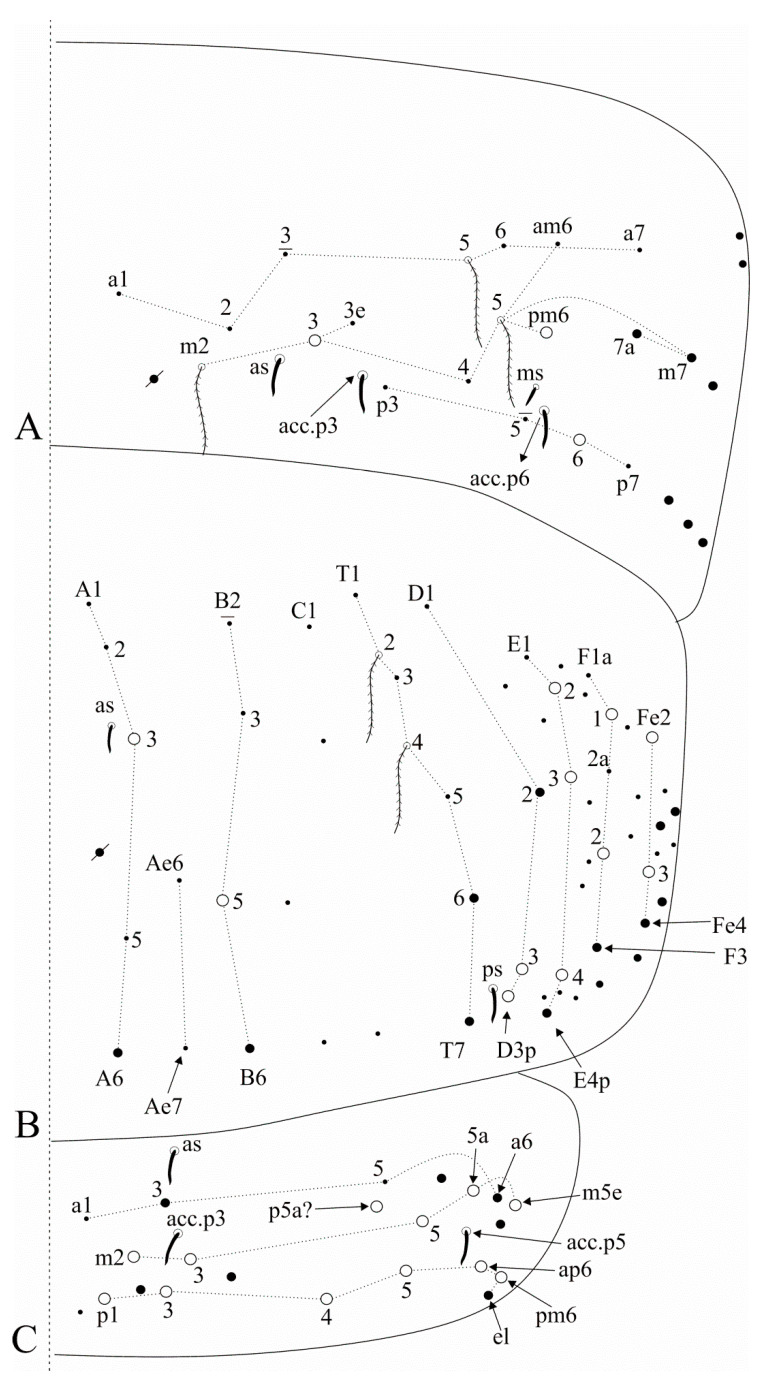
*Falcomurus hilli* sp. nov. trunk dorsal chaetotaxy, right side: (**A**) Abd. III; (**B**) Abd. IV; (**C**) Abd. V (main chaetotaxy).

**Figure 15:**
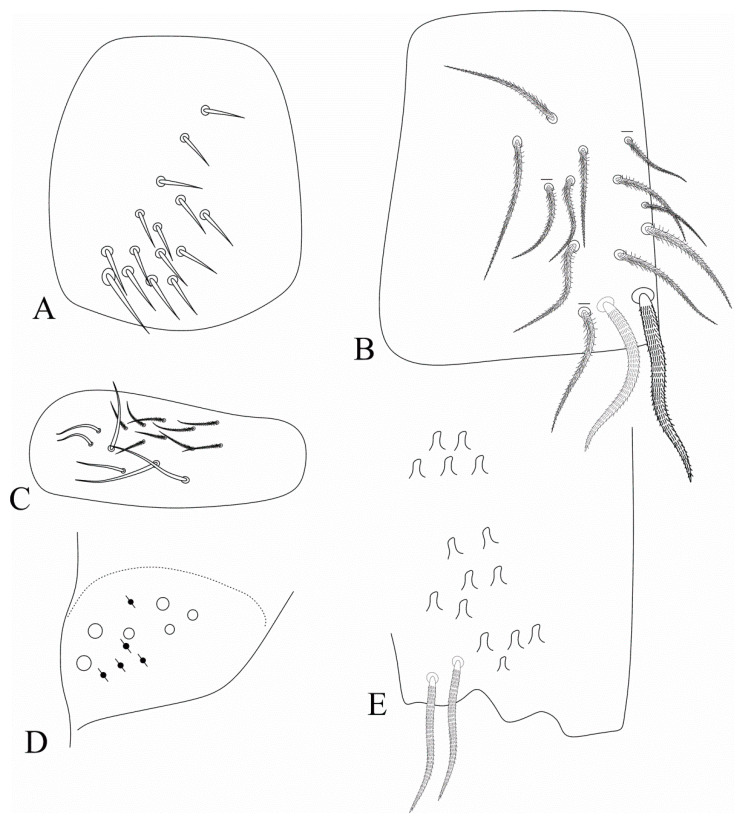
*Falcomurus hilli* sp. nov. trunk appendages: (**A**) trochanteral organ; (**B**) ventral tube anterior face, left side; (**C**) lateral flap, left side; (**D**) right manubrial plate; (**E**) ventro-distal manubrium, right side.

Figure 12, Figure 13, Figure 14 and Figure 15.

Type material: Holotype: female in slide (MVMA): Australia, the Coral Sea Islands Territory, Marion Reef, Carola Cay, intertidal zone (19°06′ S, 152°23′ E), 29.xi.1982, pitfall trap, L. Hill coll. Paratypes: five females in slides (MVMA), same data of holotype.

Other analysed material: One subadult male in slide (MVMA): Australia, the Coral Sea Islands Territory, Lihou Reef, National Native Reserve, Georgina Cay (7°25′ S, 151°40′ E), 2.viii.1984, L. Hill coll. Two juveniles in one slide (MVMA): Australia, the Coral Sea Islands Territory, Herald Cays, Southwest Cay (17°00′ S, 149°08′ E), v.2007, beach, pitfall trap, P. Greenslade coll.

Diagnosis: Ant. IV without pin projection, Ant. II subdivided in IIa and IIb, with scales. Labral **a1**–**2**, **m0**–**2** chaetae enlarged, four labral papillae present and well developed. Dorsal head with **An1a**, **Pm1**, **Pp1**, **Pp5** and **Pe3?** and mac, without **Ps2** mac, interocular area with three ciliate chaetae and one scale. Mandible apex normal, not elongated, narrow or curved. Post-labial region without smooth chaetae, including the post-labial quadrangle. Th. II with two **m4** and four central **p** mac, respectively. Abd. IV with two central mac (**A3** and **B5**). Ventral tube without scales, lateral flap with about nine ciliate and six smooth chaetae. Trochanteral organ with 14–17 chaetae. Tibiotarsi scaled proximally. Unguiculi with the outer tooth. Dorsal manubrium without short smooth chaetae, manubrial plate with six chaetae.

Description: Body length (head + trunk) of holotype = 1.54 mm; range of type series adults (*n* = 4) length = 1.54–1.66 mm; average body length of adults (*n* = 4) = 1.6 mm. Colour pattern unknown. Coarsely ciliate apically rounded or truncate scales present on Ant. I–II, head (dorsally and ventrally), dorsal trunk, legs (including proximal tibiotarsi), ventral and lateral manubrium and dens. Ventral tube unscaled.

Head (Figure 12). Antennae shorter than body, ratio antennae:body of holotype = 1:2.3; Ant. III slightly smaller than Ant. IV, antennal ratio Ant. Ia–IV of holotype = 1:5.6:1.2:9:17:20. Ant. IV and III typically annulated. Ant. IV without a subapical organite, pin projection or any clear differentiated apical sens, only with normal sens and ciliate chaetae. Ant. III sense organ with normal (not swollen) sensory rods, three guard sensilla plus at least five surrounding sens of different sizes (Figure 12A). Ant. II subdivided in IIa and IIb, IIb with few scales, dorsally with two apical sensory rods, ventrally with a single long smooth chaeta on the central region, plus ordinary ciliate chaetae and sens with different sizes and shapes (Figure 12B,C). Four labral papillae present and well developed, lateral papillae large and well marked (Figure 12D). Prelabral chaetae smooth and longer than the labral ones, labral **a1**–**2** and **m0**–**m2** enlarged, **m** series slightly longer than other labral chaetae (Figure 12E). Eyepatches lenses A–F subequal in size, G–H smaller than the others, with four interocular elements: **s**, **t** and **p** chaetae, the latter as a mes, and **v** as a scale. Head dorsal macrochaetotaxy antennal (**An**) row with seven, including **An1a**, anterior (**A**) row with five (**A0**, **A2**–**3**, **A5**–**6**), medio-ocellar (**M**) row with two (**M1**–**2**), **M0** mic present; sutural (**S**) row with six (**S1**, **S3**–**6**), post-occipital anterior (**Pa**) row with one (**Pa5**), post-occipital medial with two (**Pm1** and **Pm3**), post-occipital posterior (**Pp**) with three (**Pp1**, **Pp3** and **Pp5**) and post-occipital external with one (**Pe3?**) mac; further details are represented in Figure 12F. Mandibles normal, not remarkably elongated, narrow or curved, similar to *F. pulukokos* sp. nov. (as in Figure 8E). Maxillae normal. Labium with six main papillae (**H** plus **A**–**E**), with 2, 0, 5, 0, 4, 4 guard chaetae respectively, papilla **E** lateral process finger-shaped and short, not reaching the papilla apex; with five proximal chaetae subequal in length (**an1**–**3**, **p2**–**3**, **an1** and **p2** slightly enlarged); labium unscaled, labial basomedial (submentum or labial triangle) and basolateral (mentum) fields with chaetae **a1**–**5**, **m2** and **e** smooth, **l2** smooth or ciliate (smooth on holotype), basomedian field with 4–6 ciliate chaetae including **M1** and **R**, basolateral **L1** ciliate, subequal in length to **l2**. Maxillary outer lobe basal chaeta acuminate, slender (not erect or spiniform) and rough, shorter than the apical one, ratio basal chaeta:apical chaeta of holotype = 1:1.2; sublobal plate with four chaeta-like appendages, subequal in size. Post-labial chaetotaxy without smooth chaetae, with 77–78 ciliate chaetae of different sizes in the holotype, post-labial quadrangle with two subequal ciliate chaetae (Figure 12G).

Trunk dorsal chaetotaxy (Figure 13 and Figure 14). Tergal S-chaetotaxy from Th. II to Abd. V as 2,2|1,3,3,+,3 for sens, and 1,0|1,0,1,0,0 for ms. Th. II, excluding anterior collar, with one anterior (**a5**), three medial (**m4**–**4i** and a multiple of **m7p** row far from the collar) and 7 posterior (**p1**–**3**, **p6**–**6e**) mac (Figure 13A). Th. III with two anterior (**a5**–**6**), one medial (**m6**) and four posterior (**p1**–**3**) mac (Figure 13B). Abd. I lacking mac, **p5** mic present (Figure 13C). Abd. II with three medial (**m3**–**3e**, **m5**) mac (Figure 13D). Abd. III with two medial (**m3** and **pm6**) and one posterior (**p6**) mac, lateral tergum with at least six extra chaetae (Figure 14A). Abd. IV with two central (**A3** and **B5**) and nine lateral (**D3**–**3p**, **E2**–**4**, **F1**–**2** and **Fe2**–**3**) mac, **as** sens internal to **A3**, **ps** nearby **D3p**, central region with several long sens (not represented in the drawing), lateral tergum with at least 16 mic and five mes without clear homologies (Figure 14B). Ratio Abd. III:IV of holotype 1:1.54. Abd. V main chaetotaxy with five medial (**m2**–**3**, **m5**–**5e**), five posterior (**p1**, **p3**–**ap6**) plus **p5a?** and **pm6** mac and several secondary chaetae (only few represented in the drawing), sensillar pattern similar to *Heteromurus* (as in [3,8]) (Figure 14C). More details on the trunk’s idio and S-chaetotaxy are represented in Figure 13 and Figure 14.

Trunk appendages (Figure 15). Trochanteral organ with 14–17 spine-like smooth chaetae (Figure 15A). Foot complex as in *F. pulukokos* sp. nov. (Figure 11A). Ventral tube anteriorly without smooth chaetae, with two distal mac (the inner larger than the outer one), plus 9–12 ciliate chaetae of different sizes (Figure 15B). Posterior face not entirely clear, with at least 16 lateral ciliate chaetae and a pair of centro-distal long smooth chaetae. Lateral flaps with about nine ciliate and six smooth chaetae each, three smooth chaetae larger than the others (Figure 15C). Tenaculum corpus with a single clearly ciliate chaeta, each ramus with four teeth. Manubrium and dens lacking smooth chaetae of any size. Manubrial plate with six chaetae, the two internal slightly larger than the others, plus five small pseudopores (Figure 15D). Ventro-distal manubrium with two inner ciliate chaetae plus about 10 scales (Figure 15E). Dorso-proximal dens with a typical sinuous ciliate mac (Figure 11F), mucro apical tooth larger than the basal one, mucronal spine typically absent (Figure 11G).

Etymology: The species honours the entomologist Lionel Hill, who collected the type material.

Remarks: The main useful features to separate *Falcomurus* species are the subdivision of Ant. II, mandibles apex, labral papillae morphology, posterior dorsal head chaetotaxy, presence of smooth chaetae in the post-labial region, Th. II central chaetotaxy, presence of **A6** mac on Abd. IV, presence of the outer tooth on the unguiculi and manubrial dorsal chaetotaxy. In that regard, *F. chilikaensis* is unique due the presence of **Ps2** mac on the dorsal head and Th. II with a complex central macrochaetotaxy, with three **m4** mac and seven mac on the **p1**–**3** group, unguiculi without the outer tooth and dorsal manubrium with short smooth chaetae [15]. This species also combines an undivided Ant. II, post-labial quadrangle with smooth chaetae and **A6** mac present on Abd. IV. Unfortunately, Mandal’s description did not provide data on the mandibles’ apex and labral papillae.

As noted by [17], the main characteristics to distinguish *F. halophilus* comb. nov. from *F. litoreus* comb. nov. are the degree of labral papillae development (fully developed in *F. halophilus* comb. nov. vs. weakly developed in *F. litoreus* comb. nov.), posterior head macrochaetotaxy (with **Pm1** and **Pp5** mac in *F. halophilus* comb. nov. vs. without in *F. litoreus* comb. nov.) and Abd. IV central macrochaetotaxy (with three mac in *F. halophilus* comb. nov. vs. two in *F. litoreus* comb. nov.). The author also remarked on the presence of scales on Ant. II in the latter species (vs. absence in *F. halophilus* comb. nov.); however, our analysis of the new species showed the number of scales in this segment is reduced, which could be easily overseen due to their fall. Some unknown or unclear features of *F. halophilus* comb. nov., such as the presence of a ventral tube and tibiotarsi scales, ventral tube lateral flap’s chaetotaxy, the exact number of trochanteral organ smooth chaetae, among other characteristics, could be useful to better compare this species with its other congeners.

*Falcomurus pulukokos* sp. nov. and *F. hilli* sp. nov. are the only species in the genus with the Ant. II subdivided [15,17]. They also differ from Mari-Mutt’s species by their normal mandible apex (curved in *F. halophilus* comb. nov. and *F. litoreus* comb. nov.) and interocular region with four elements, three chaetae and one scale (five in *F. halophilus* comb. nov. and *F. litoreus* comb. nov.). The new species differ from each other by their Ant. III sensory rods (swollen in *F. pulukokos* sp. nov., normal in *F. hilli* sp. nov.); labral papillae (four in *F. hilli* sp. nov. and two in *F. pulukokos* sp. nov.); **m1**–**0** labral chaetae enlarged and dorsal head **Pp5** as mac only in *F. hilli* sp. nov.; post-labial chaetotaxy without smooth chaetae on *F. hilli* sp. nov. (with four in *F. pulukokos* sp. nov.); and ventral tube’s lateral flaps without ciliate chaetae only in *F. pulukokos* sp. nov.

More comparisons among the species are presented in Table 1. An identification key to the *Falcomurus* taxa based on our comparisons is presented in the next section.

### 3.7. Identification Key and Distribution of Falcomurus Species

1. Dorsal head with **Ps2** mac, Th. II with three **m4** and seven **p** central mac (from **p1**–**3** group), manubrium dorsally with short smooth chaetae, unguiculi lacking the outer tooth … *F. chilikaensis* Mandal, 2018 (India);

− Dorsal head without **Ps2** mac, Th. II with two **m4** and four **p** central mac (from **p1**–**3** group), manubrium dorsally without smooth chaetae, unguiculi with the outer tooth … 2;

2 Ant. II undivided, mandibles apex curved, interocular field with five elements … 3;

− Ant. II subdivided in IIa and IIb, mandibles apex normal, interocular field with four elements … 4;

3 Labral papillae well developed, dorso-posterior head with **Pm1** and **Pp5** mac, Abd. IV with three central mac (**A3**, **A6** and **B5**) … *F. halophilus* (Mari-Mutt, 1985) comb. nov. (Papua New Guinea, Main Island);

− Labral papillae underdeveloped, dorso-posterior head without **Pm1** and **Pp5** mac, Abd. IV with two central mac (**A3** and **B5**) … *F. litoreus* (Mari-Mutt, 1985) comb. nov. (The Philippines, Mindoro Island);

4 Labrum with four papillae, dorso-posterior head with **Pp5** mac, post-labial anterior region and post-labial quadrangle without smooth chaetae, trochanteral organ with 14–17 smooth chaetae, ventral tube’s lateral flap with smooth and ciliate chaetae … *F. hilli* sp. nov. (Australia, the Coral Sea Islands Territory);

− Labrum with two papillae, dorso-posterior head without **Pp5** mac, post-labial anterior region and post-labial quadrangle with smooth chaetae, trochanteral organ with more than 20 smooth chaetae, ventral tube’s lateral flap only with smooth chaetae … *F. pulukokos* sp. nov. (Australia, Cocos and Keeling West Island).

## 4. Discussion

### 4.1. On Falcomurus Species Morphology

The overall morphology of *Falcomurus* species is well conserved in its five species. Regarding the three species we could analyse in detail, namely, *F. litoreus* comb. nov., *F. pulukokos* sp. nov. and *F. hilli* sp. nov., not only their dorsal macrochaetotaxy is remarkably similar, but also other elements of the idio and sensillar chaetotaxy as well, with the chaetae in similar positions in the three species. Some mac on the Abd. IV and V interchange to mes in different species, such as **A6** and **D2** on Abd. IV and **m5a** and **a6** on Abd. V, differences we could better track studying the alveoli of the chaetae. Nevertheless, such differences are minor considering the general dorsal chaetotaxy of the genus. Other important features shared by the three species that may be constant within *Falcomurus* are the sublobal plate with four chaetae-like appendages; labial papillae guard chaetae formula; labial basal fields scaleless, with a variable set of ciliate chaetae on the labial triangle; foot complex overall morphology (with the exception of the unguicular outer tooth absent in *F. chilikaensis*); and the presence of two pairs of ciliate chaetae on the ventral manubrium (unclear in *F. litoreus* comb. nov.).

On the other hand, some variable characteristics caught our attention, especially the presence of subdivisions on Ant. II seen in *F. pulukokos* sp. nov. and *F. hilli* sp. nov. Although the subdivision of the Ant. II is a useful taxonomic character to delimit genera among the Orchesellidae, it proved to be variable within genera and even species, such as within *Dicranocentrus* and *Nothobrya* Arlé, 1961 [6,29,39]. Other variable features between the species are the scales on Ant. II, anterior ventral tube and proximal tibiotarsi. As previously remarked, the scales on Ant. II may be an unreliable feature by which to compare *Falcomurus* species, as they are reduced in number in *F. pulukokos* sp. nov. and *F. hilli* sp. nov., and were only seen in few specimens. The same occurs for the tibiotarsal scales, and while *F. litoreus* comb. nov. has several proximal scales on tibiotarsi, *F. hilli* sp. nov. only has a few, seen in few specimens. However, the scaled ventral tube of *F. litoreus* comb. nov. is clearly seen as there are several scales from the base to the apex, and if they fall, their alveoli could still be reliable to define the species.

### 4.2. On Falcomurus Affinities

As noted by [17] and partially by [15], the dorsal chaetotaxy of *Falcomurus* is remarkably similar to that of *Heteromurus s. str.* Among the Heteromurini, at least two other taxa show a clear reduced dorsal chaetotaxy, *Heteromurtrella* and *Verhoeffiela*, although the dorsal chaetotaxy of *Dicranocentrus* is variable, and some species show marked reductions in the number of mac also [8,12,29,33,37,38,40]. Excluding *F. litoreus* comb. nov. and *F. halophilus* comb. nov. from *Dicranocentrus*, no other species of the genus is devoid of mac on Abd. I. As pointed out in [17], the only species with a similar reduction in dorsal macrochaetotaxy in *Dicranocentrus* is *D. singularis* Mari-Mutt and Bhattacharjee, 1980 [40], from India, which is also devoid of the **S0** mac on the dorsal head. Even so, this species presents 2 + 2 mac on the Abd. I [40] (pp. 168–169, Figure 33). Although *Falcomurus* has the Ant. II undivided in part of its species and the mucronal spine absent, such features were also recorded in *Dicranocentrus* taxa from India: in *D. indicus* Bonet, 1930 [41] sensu Yosii [42] the mucronal spine can be present or absent; while *D. pilosus* Mari-Mutt and Bhattacharjee 1980 [40], do not show a clear division of the Ant. II, which may occur abnormally in other species of the genus as well [29]. The sinuous dental mac seen in *Falcomurus* may also have a parallel within *Dicranocentrus*. At least *D. indicus*, *D. janetscheki* Yosii, 1971 [43], from Nepal, and *D. javanus* Yoshii and Suhardjono, 1989 [5], from Java, show one or two pairs of ciliate mac on the proximal dens. However, in the three species, the chaetae are erect and blunt, somewhat different from *Falcomurus*. Furthermore, the dorsal macrochaetotaxy of these three latter species is more complex than in the late genus.

The dorsal head **Ps2** mac seen in *F. chilikaensis* was only observed previously in one other genus of Heteromurini, *Pseudodicranocentrus* [8,33]. However, this genus differs from *Falcomurus* by: the prelabral chaetae bifurcate, dorsal head **A1** and **S0** mac present, and a clearly more complex trunk macrochaetotaxy, with 4–5 mac on Abd. I [8,33]. So, a close relationship between *Pseudodicranocentrus* and *Falcomurus* is unlikely, and the **Ps2** mac seen in *F. chilikaensis* may have arisen independently from the former genus.

*Heteromurus s. str.* and *Verhoeffiela* are closely related, and recent studies strongly support *Verhoeffiela* as an ingroup of *Heteromurus*, possibly being polyphyletic [44,45]. They will be herein be treated combined as *Heteromurus s. lat.*, as in [8]. Accordingly, with the overall similarity of chaetotaxy of *Falcomurus* with *Heteromurus s. lat.*, other features may support a closer relationship between them, like the absence of the mucronal spine (seen in part of *Heteromurus s. str.*), Ant. III annulated (as in *Verhoeffiela*), and more importantly, the sensillar pattern of Abd. V with three sens displaced anteriorly. This last character, also noted by [15], may hold a strong phylogenetic signal within the Heteromurinae, as proposed by [8], and puts *Falcomurus* closer to *Heteromurus s. lat.*, possibly as an ingroup of the later.

Lastly, further studies on Heteromurinae phylogenetics are needed to clarify the correct position of *Falcomurus* within the subfamily. At this time, since no species of the genus have yet been included in molecular phylogenies, it is not clear if it is an ingroup of *Dicranocentrus* or *Heteromurus s. lat.*, an extant genus from a transitional group between them, or even an independent genus of Heteromurinae separated from both.

### 4.3. On Falcomurus Habitat, Ecology and Distribution

**Figure 16:**
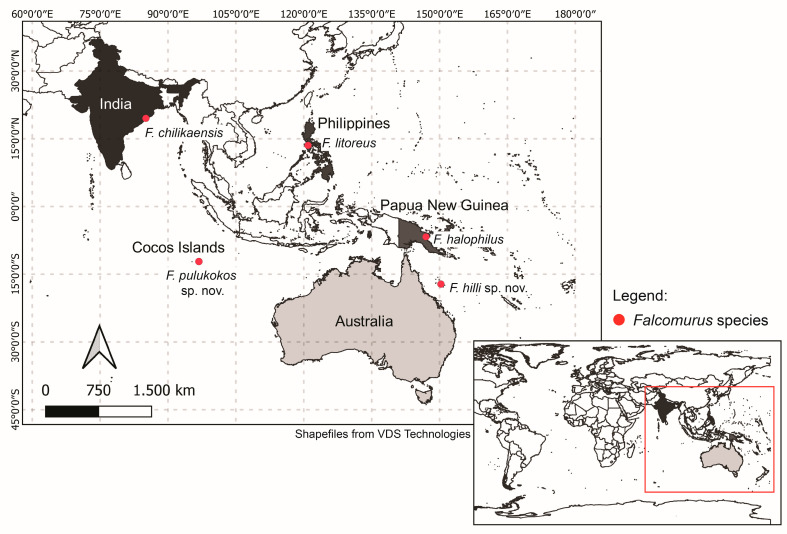
Distribution of *Falcomurus* species.

The genus *Falcomurus*, with two new species and two transferred from *Dicranocentrus*, now contains five species. Their distribution, as currently known, extends from the Bay of Bengal on the east coast of the Indian subcontinent to the Coral Sea and Philippines (Figure 16). All five species have only been found in marine littoral habitats and in the upper intertidal zone of sandy beaches, just below high tide mark [15,17]. They appear to be exclusive to this habitat. Thus, *Falcomurus* is the only genus of Heteromurinae entirely restricted to marine littoral environments [46].

The marine littoral habitat has been largely neglected by springtail taxonomists, with the main exception being J-M Thibaud. However, Thibaud has not been active in south-east Asia and the Pacific so we suspect the genus distribution is wider than currently known. In fact, the current genus distribution suggests it may also be present in Indonesia (Figure 16).

Marine littoral fauna can be highly diverse and comprises genera not found in any other habitat, as in the case of *Falcomurus*; for instance, on Barrow Island (Western Australia), in the intertidal zones of four beaches of different types, including mud flats with mangroves, coral sand, rocky shores and high-velocity, steep sandy beaches. Altogether, 14 species of springtails were found here belonging to 11 genera. It was assumed that all species were intertidal specialists, while seven of the genera were also restricted to this habitat [47]. A different suite of species was found on four types of beaches.

## 5. Conclusions

After this revision, there are now five species of *Falcomurus*. Their morphology is mostly conserved, and some aspects of the dorsal head posterior, post-labial, Th. II and Abd. IV chaetotaxy are the most useful features to separate them from each other. The overall morphology of *Falcomurus* resembles both *Dicranocentrus* and *Heteromurus s. lat.*, but the Abd. V sensillar chaetotaxy and Abd. I lacking mac may put the genus closer to the latter, as they differ from *Dicranocentrus*. On the other hand, the presence of a proximal falcate mac on dens may be exclusive of *Falcomurus*. With the description of two new species and the transfer of *D. litoreus* and *D. halophilus* to *Falcomurus*, the genus distribution has extended from the Bay of Bengal (India) to the Coral Sea (Australia) and Philippines. These new species records are consistent in habitat (the marine littoral environments) with that of the type species, *F. chilikaensis*.

## Figures and Tables

**Table 1:** Diagnostic characters of *Falcomurus* species *.

Characters/Species		*chilikaensis*	*halophilus* comb. nov.	*litoreus* comb. nov.	*pulukokos* sp. nov.	*hilli* sp. nov.
Ant. IV pin projection		+	−	?	−	−
Ant. II subdivision		−	−	−	+	+
Ant. II scales		?	−	+	+	+
Labral papillae		?	2 dev.	2 undev.	2 dev.	4 dev.
Labral **a2** chaeta		N	E	E	E	E
Labral **m2** chaeta		N	N ?	N	E	E
Labral **m0–1** chaetae		N	N ?	N	N	E
Mandible apex		?	curved	curved	normal	normal
Interocular chaetotaxy		?	4 ch, 1 sc or	4 ch, 1 sc or	3 ch, 1 sc	3 ch,1 sc
			5 ch	3 ch, 2 sc		
Dorsal head mac	**An1a**	− ?	−	−	+	+
	**Ps2**	+	−	−	−	−
	**Pm1**	− ?	+	−	+	+
	**Pp1**	− ?	+	+/−	+	+
	**Pp5**	− ?	+	−	−	+
PLQ smooth chaetae		+	−	−	+	−
PL anterior smooth chaetae		?	+	+	+	−
Th. II mac	**m4** group	3	2	2	2	2
	**p1–3** group	7	4	4	4	4
Abd. IV central mac		3 **	3	2	2	2
Trochanteral organ chaetae		16–18	27–32 ?	27–32	>20	14−17
Tibiotarsi scales		+	?	+	−	+
Unguiculus outer tooth		−	+	+	+	+
Ventral tube scales		− ?	?	+	−	−
Lateral flap chaetae		18 sm	?	6 sm, 26 cl	22 sm	6 sm, 9 cl
Manubrial smooth chaetae		+ ***	−	−	−	−
Manubrial plate chaetae		?	?	11−13	7−8	6

Legends: + = present; − = absent; > = more than; ? = unknown/unclear; dev = developed (clearly visible); undev = underdeveloped (hardly visible); N = normal; E = enlarged; ch = chaeta(e); sc = scale(s); sm = smooth; cl = ciliate; PL = post-labial; PLQ = post-labial quadrangle. * Colour patterns are presented in the species diagnoses/descriptions; ** see the remarks on the species; *** manubrial smooth chaetae on *F. chilikaensis* are short, different from most Heteromurinae [17] (p. 81, Figure 15). Data on *F. chilikaensis*, *F. halophilus* comb. nov. and *F. litoreus* comb. nov. are based on original descriptions and our revision. Chaetae labels (including rows/series) and labial papillae are marked in bold.

## Data Availability

All data is contained within the article. All biological material is deposited at MVMA as previously stated.
